# SNMP1 is critical for sensitive detection of the desert locust aromatic courtship inhibition pheromone phenylacetonitrile

**DOI:** 10.1186/s12915-024-01941-x

**Published:** 2024-07-08

**Authors:** Joris Lehmann, Yannick Günzel, Maryam Khosravian, Sina Cassau, Susanne Kraus, Johanna S. Libnow, Hetan Chang, Bill S. Hansson, Heinz Breer, Einat Couzin-Fuchs, Joerg Fleischer, Jürgen Krieger

**Affiliations:** 1 Department of Animal Physiology, Institute of Biology/Zoology, Martin Luther University Halle-Wittenberg, Halle (Saale), Germany; 2 Department of Biology, University of Konstanz, Konstanz, Germany; 3 Centre for the Advanced Study of Collective Behaviour, University of Konstanz, Konstanz, Germany; 4 Department of Collective Behavior, Max Planck Institute of Animal Behavior, Konstanz, Germany; 5 International Max Planck Research School for Quantitative Behavior, Ecology and Evolution From Lab to Field, Konstanz, Germany; 6 Department of Evolutionary Ecology, Max Planck Institute for Chemical Ecology, Jena, Germany; 7 Shenzhen Branch, Guangdong Laboratory for Lingnan Modern Agriculture, Synthetic Biology Laboratory of the Ministry of Agriculture and Rural Affairs, Agricultural Genomics Institute at Shenzhen, Chinese Academy of Agricultural Sciences, Shenzhen, China; 8 Department of Physiology (190V), Institute of Biology, University of Hohenheim, Stuttgart, Germany

**Keywords:** Insect olfaction, Sensory neuron membrane protein 1, *Schistocerca gregaria*, Pheromone communication, Antenna, Antennal lobe

## Abstract

**Background:**

Accurate detection of pheromones is crucial for chemical communication and reproduction in insects. In holometabolous flies and moths, the sensory neuron membrane protein 1 (SNMP1) is essential for detecting long-chain aliphatic pheromones by olfactory neurons. However, its function in hemimetabolous insects and its role for detecting pheromones of a different chemical nature remain elusive. Therefore, we investigated the relevance of SNMP1 for pheromone detection in a hemimetabolous insect pest of considerable economic importance, the desert locust *Schistocerca gregaria*, which moreover employs the aromatic pheromone phenylacetonitrile (PAN) to govern reproductive behaviors.

**Results:**

Employing CRISPR/Cas-mediated gene editing, a mutant locust line lacking functional SNMP1 was established. In electroantennography experiments and single sensillum recordings, we found significantly decreased electrical responses to PAN in SNMP1-deficient (SNMP1^−/−^) locusts. Moreover, calcium imaging in the antennal lobe of the brain revealed a substantially reduced activation of projection neurons in SNMP1^−/−^ individuals upon exposure to PAN, indicating that the diminished antennal responsiveness to PAN in mutants affects pheromone-evoked neuronal activity in the brain. Furthermore, in behavioral experiments, PAN-induced effects on pairing and mate choice were altered in SNMP1^−/−^ locusts.

**Conclusions:**

Our findings emphasize the importance of SNMP1 for chemical communication in a hemimetabolous insect pest. Moreover, they show that SNMP1 plays a crucial role in pheromone detection that goes beyond long-chain aliphatic substances and includes aromatic compounds controlling reproductive behaviors.

**Supplementary Information:**

The online version contains supplementary material available at 10.1186/s12915-024-01941-x.

## Background

Under given environmental conditions, swarm-forming locusts, such as the desert locust *Schistocerca gregaria* and the migratory locust *Locusta migratoria*, undergo a transition from a sedentary and solitarious to a group-living (gregarious) phase. This phase transition entails a faster reproduction cycle and aggregation, culminating in the formation of large swarms that cause tremendous damage to natural vegetation and crops across large landscapes of Africa and Asia [1–3]. In locusts, phase transition, reproduction, and aggregation involve the integration of visual, tactile, and olfactory information [3–6]. Among the olfactory signals, pheromones — as well as chemicals in locust feces — have been reported to induce and control reproductive behavior and aggregation [3, 4, 7–10]. So far, only a few locust pheromones have been identified. In *Locusta migratoria*, phenylacetonitrile (PAN) has been described as an anti-cannibalistic pheromone under crowded conditions [11]. In the desert locust *S. gregaria*, PAN serves a different function [7]. In this species, it is only released in high concentrations by sexually mature gregarious males, thereby signaling male identity and preventing courtship with individuals of the same sex. Moreover, male-released PAN hides a receptive female during male mate guarding and protects her against courtship by male rivals, thus acting as a courtship inhibition pheromone [12–14].

Locusts detect pheromones and odorants by olfactory sensory neurons (OSNs) residing in cuticular structures of the antenna termed sensilla [15, 16]. Locust OSNs are housed in three morphologically distinct olfactory sensilla types, i.e., sensilla basiconica (each of them harbors 20–50 OSNs), sensilla trichodea (1–3 OSNs), and sensilla coeloconica (1–4 OSNs) [17]. Among these sensilla types, PAN is detected by OSNs of basiconic sensilla, whereas it does not activate sensory cells in sensilla trichodea or sensilla coeloconica [15].

Like in other insects, the axonal processes of locust OSNs project to the antennal lobe (AL), the first odor-processing center in the brain. In neuropil structures of the AL called glomeruli, the axons of OSNs synapse with projection neurons (PNs) that are the sole output cells of the AL. PNs convey the olfactory information via their axons to higher-order brain centers, including the mushroom body [18–21]. As a distinctive feature of the locust olfactory system, the axon of an OSN is often branched and innervates several of the more than 1000 small glomeruli [22]. In addition, each of the PNs connected to these glomeruli innervates up to 20 glomeruli [18, 19, 23–25].

To detect odorants and pheromones, insect OSNs are equipped with olfactory receptors mainly belonging to the families of odorant receptors (ORs) and ionotropic receptors (IRs) [26–30]. OSNs employing members of the OR family co-express the odorant receptor co-receptor ORCO [31]. ORCO forms heteromeric cation channels with ORs that are activated upon binding appropriate odorous or pheromonal ligands [32–34]. In addition, subsets of OR-positive OSNs co-express the so-called “sensory neuron membrane protein 1” (SNMP1), a protein predominantly expressed in the antenna and localized in the dendrites of OSNs that were found to be crucial for pheromone detection [35–42]. SNMP1 proteins represent insect-specific members of the CD36 protein family (named after the human protein “cluster of differentiation 36”) that comprises vertebrate and invertebrate proteins with remarkable functional versatility, acting as receptors and transporters for long-chain fatty acids and lipoproteins or having functions in immune signaling and cell adhesion [41, 43–46]. So far, the specific function of SNMP1 in OSNs has only been examined in holometabolous insects, mainly in the fly *Drosophila melanogaster* and in some moth species [35, 37–39, 47]. In *Drosophila melanogaster*, SNMP1 is vital for the detection of the male-specific courtship inhibition pheromone cis-vaccenyl acetate (cVA), a long-chain lipid acetate ester received via OSNs expressing the OR type OR67d [35, 37]. Likewise, in the moth species *Helicoverpa armigera* and *Heliothis virescens*, SNMP1 is critical for the detection of female-released long-chain sex pheromones, such as (Z)-11-hexadecenal and (Z)-9-hexadecenal [38, 47]. As a characteristic feature of the CD36 family members, insect SNMP1 proteins comprise two predicted transmembrane regions and a large extracellular domain [40]. Homology-based modeling of the *Drosophila* SNMP1 ectodomain using a mammalian CD36 family member as a template suggested that the large extracellular domain might form a tunnel-like structure. Hence, the ectodomain of SNMP1 could serve as a funnel transporting ligands in the extracellular lymph to cognate pheromone-detecting ORs in the dendritic membrane [36].

While SNMP1 function has been studied in holometabolous insects (Endopterygota), its specific role in the olfactory system of hemimetabolous insects (Exopterygota) is unknown. In the antenna of the hemimetabolous desert locust, we previously observed SNMP1 expression in a substantial subpopulation of OR-expressing OSNs as well as in some support cells [48, 49]. Across the antennal OSNs, SNMP1 is co-expressed with a considerable number of different OR types. In fact, out of 83 *S. gregaria* OR types analyzed (~ 70% of the known OR repertoire in this species), 33 were found to be co-expressed with SNMP1 [50, 51]. These findings suggest a pivotal role for SNMP1 in *S. gregaria* olfaction and indicate an interplay with various OR types. It is, however, currently unclear whether SNMP1 might also contribute to detecting pheromonal substances in locusts, in particular the behaviorally relevant pheromone PAN. Remarkably, this aromatic pheromone is structurally quite different from the long-chain aliphatic pheromonal compounds of *Drosophila melanogaster* and moths that require SNMP1 for their detection [35, 37, 38, 47]. Against this background, we set out to inspect a potential role of SNMP1 in detecting PAN in the desert locust. To this end, we generated desert locusts deficient in SNMP1 (SNMP1^−/−^) utilizing the CRISPR/Cas gene editing technique [52]. Subsequently, the impact of SNMP1 deficiency on the antennal detection of PAN and on pheromone-controlled behavior was studied by comparing PAN-induced responses in SNMP1^−/−^ animals and wild-type (WT) conspecifics using electrophysiological and behavioral approaches. Moreover, performing calcium imaging with PNs in the AL, we analyzed the consequences of the lack of functional SNMP1 in OSNs on PAN-induced neuronal activity in the first relay station for olfactory information in the brain.

## Results

### Generation of desert locusts mutant for SNMP1

In order to generate an SNMP1-deficient mutant locust line (SNMP1^−/−^) using the CRISPR/Cas technique, we set out to identify suitable guide RNA sequences. Analyzing a published draft version of the *S. gregaria* genome revealed that the coding sequence of SNMP1 is derived from nine different exons (Additional file 1: Fig. S1). As guide RNA, a fragment of 20 nucleotides located upstream of a protospacer adjacent motif (PAM) site residing approximately in the center of exon 4 was chosen.

**Fig. 1:**
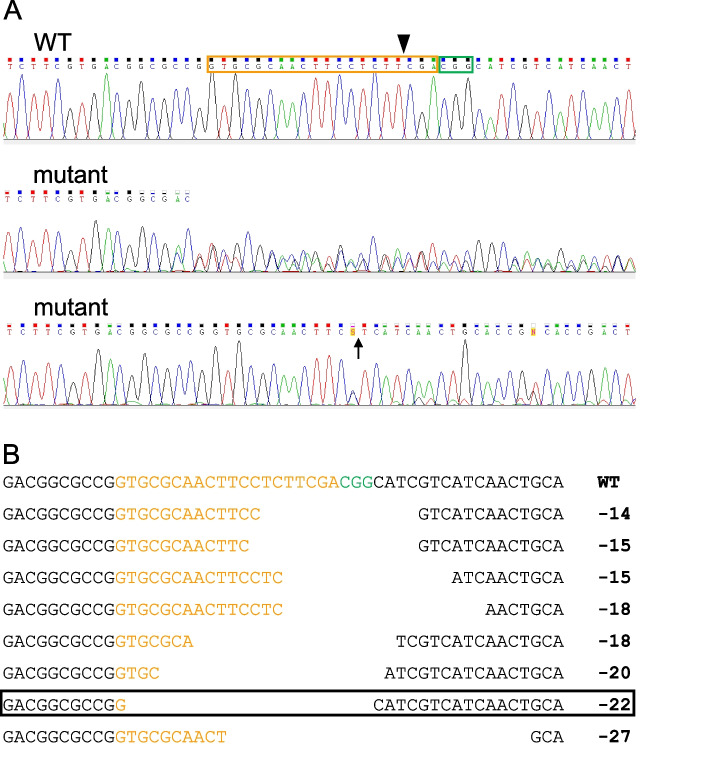
Identification of mutations in exon 4 of the *S. gregaria* SNMP1 gene. **A** Sequencing chromatograms of PCR products amplified from exon 4 of the SNMP1 gene. The region corresponding to the guide RNA (orange box) and the PAM site (green box) is depicted for WT and mutant animals of the G0 generation. The putative site of the Cas9-induced double-strand break (3 bp upstream of the PAM site) is denoted by the arrowhead in the WT sequence. The arrow in the lower panel marks the site of a 15-bp deletion. Chromatograms were visualized using the Chromas software (Technelysium, South Brisbane, Australia). **B** WT and mutant nucleotide sequences of exon 4 from individuals of the G1 generation. The region corresponding to the guide RNA (orange) and the PAM site (green) is shown. The number of deleted nucleotides is indicated on the right for each mutation. The mutation (22-bp deletion) carried by the individuals chosen to generate the homozygous mutant line SNMP1^−/−^ is boxed

Following injection of the single-guide RNA and the Cas9 protein in several dozens of freshly laid eggs, we obtained a total of 19 adult locusts (males and females; generation G0) that could be scrutinized for the presence of mutations in exon 4 of the SNMP1 gene (from most of the eggs, no nymphs hatched, or the nymphs died before reaching the adult stage). Genomic DNA from these G0 adult animals was prepared and utilized as a template in PCR reactions along with a primer pair matching the 5′ and the 3′ end of exon 4 of the SNMP1 gene. For all of the 19 individuals examined, Sanger sequencing of the resulting PCR product led to an electropherogram different from WT controls, indicating that they were mutants for SNMP1 (Fig. 1A). For most of these animals, sequencing of the PCR product yielded an electropherogram from which no clearly readable nucleotide sequence could be derived (exemplarily depicted in the middle panel in Fig. 1A). In these cases, it seemed that different sequences were overlaid, suggesting that the animals were heterozygous, i.e., either endowed with WT and mutant alleles or only with different mutant alleles. For four individuals, however, no clear overlay of different sequences was immediately recognizable (exemplarily shown in the lower panel in Fig. 1A); yet, a more detailed analysis disclosed deletions of 15 or 18 nucleotides in the region corresponding to the guide RNA and the PAM site (data not shown). Since these deletions do not induce a shift in the reading frame, the relevant animals were not considered further. The other (putative) mutants were individually mated with WT conspecifics of the opposite sex, and the resulting offspring (generation G1) was screened for mutations in exon 4 of the SNMP1-encoding gene. Therefore, utilizing genomic DNA from 41 G1 individuals, PCR reactions with the aforementioned primer pair for exon 4 of the SNMP1 gene were conducted. Subsequent to cloning and sequencing of the PCR products, ten different mutations were found in the region of exon 4 that corresponds to the guide RNA and the PAM site (some of them are exemplarily depicted in Fig. 1B). All of these mutations were characterized by deletions ranging from 14 to 94 base pairs (bp); none of them contained nucleotide substitutions or insertions. To establish a homozygous line mutant for SNMP1, animals with a deletion of 22 bp in exon 4 were chosen for two reasons. First, in contrast to some others, this mutation should cause a shift in the reading frame, leading to a severely truncated protein lacking more than half of the WT protein (Additional file 2: Fig. S2). And second, several animals carrying this mutation were identified, including males and females that successfully reproduced when mated with each other. Finally, from the resulting G2 generation, only animals that were homozygous for this 22-bp deletion (SNMP1^−/−^) were used for further mating.

### Analysis of SNMP1 expression in the SNMP1^−/−^ mutant line

To assess whether the 22 bp deleted from exon 4 of the SNMP1 gene are also absent from the corresponding mRNA, antennal RNA from SNMP1^−/−^ animals was reversely transcribed into cDNA that was used in subsequent PCR approaches with a sense primer matching exon 3 and a reverse primer matching exon 6 of the SNMP1 gene. Sequencing of the amplicon revealed that the antennal cDNA indeed lacked the 22 bp deleted from exon 4 of the SNMP1 gene in mutants, while further mutations were not observed (data not shown). Due to the 22-nucleotide deletion in the mRNA leading to a frameshift, a premature termination codon was found, which substantially truncates the amino acid sequence of SNMP1 in mutant animals (Additional file 2: Fig. S2).

**Fig. 2:**
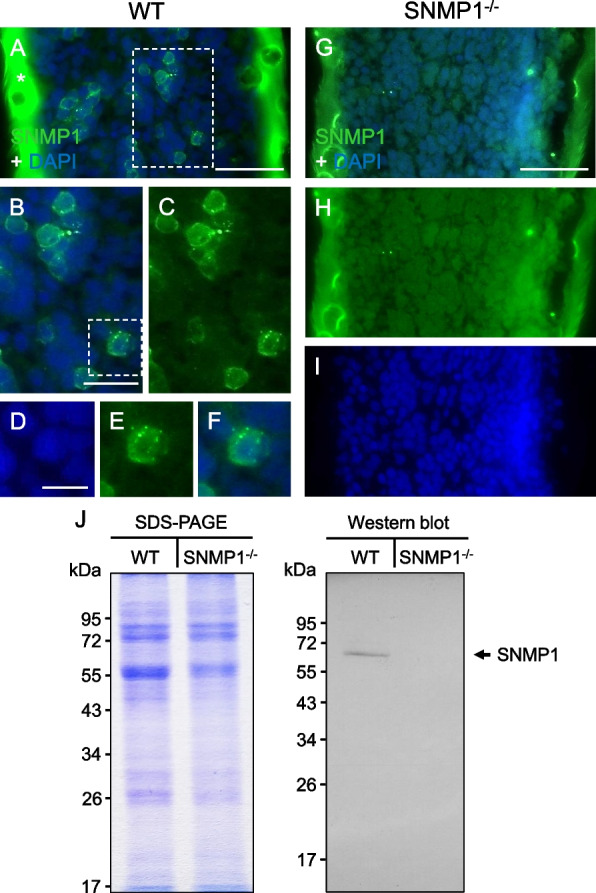
SNMP1 immunoreactivity is absent from the antennae of SNMP1^−/−^ desert locusts. **A** Immunohistochemical staining with an SNMP1-specific antibody (green) on a longitudinal section through the antenna of an adult WT male that was counterstained with DAPI (blue). Green immunostaining marks several SNMP1-positive cells. The green color of the cuticle (denoted by the asterisk) is due to autofluorescence. **B,C** Higher magnifications of the boxed area in **A** (in **C**, only the green fluorescence channel is shown). **D**–**F** High magnification images of the SNMP1-positive cell boxed in **B**. The blue (**D**), the green (**E**), and the overlay of both channels (**F**) are displayed. **G** No labeled cells were detectable upon immunostaining with the SNMP1 antibody on a longitudinal section through the antenna from an adult male of the SNMP1^−/−^ line. **H–I** The green (**H**) and the blue (**I**) channels are depicted separately for the area shown in **G**. The results shown in **A**–**I** are representative of three independent experiments; staining of sections from WT and SNMP1^−/−^ desert locusts were conducted simultaneously. Scale bars: **A** and **G** = 50 μm; **B** = 20 μm; **D** = 10 μm. **J** SDS-PAGE (stained with Coomassie blue, left panel) and Western blot analysis (right panel) with 10 µg of protein fractions prepared from the antennae of WT or SNMP1.^−/−^ desert locusts (males and females). Immunodetection with the antibody against SNMP1 labeled a band with the predicted molecular mass of SNMP1 (~ 57 kDA; indicated by the arrow) only in the protein fraction derived from WT animals. The molecular mass and position of molecular weight standards used in the SDS-PAGE and the Western blot analysis are given on the left. The original images of the SDS-PAGE and the Western blot are depicted in Additional file 9: Fig. S8

**Fig. 3:**
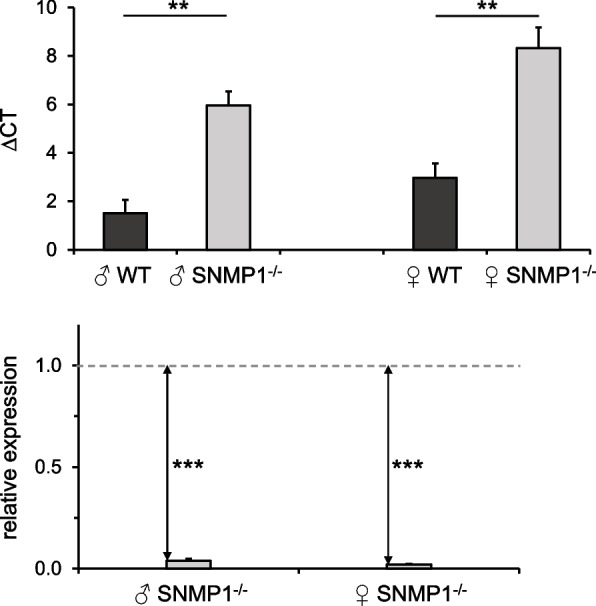
Expression of SNMP1-encoding mRNA is significantly reduced in the antenna of the SNMP1^−/−^ line. A primer pair matching exon 9 of the SNMP1 gene and antennal cDNA from male or female WT and SNMP1^−/−^ animals were used to examine the expression of SNMP1 by determining the threshold cycle (CT) via quantitative PCR. For normalization, the reference gene GAPDH served as the endogenous standard. In the upper panel, ∆CT values (CT_SNMP1_ − CT_GAPDH_) are given. The lower panel shows the relative expression for SNMP1 in the antenna of SNMP1.^−/−^ individuals, which was calculated according to Pfaffl [54] using the CT values determined. The dashed horizontal line marks a relative expression level of 1, i.e., an SNMP1 expression rate identical to that in WT antennae. The results are based on four independent experiments (for male and female animals, respectively). Error bars denote the standard error of the mean. Two-tailed *p*-values were calculated by unpaired *t*-tests (***, *p* < 0.001; **, 0.001 ≤ *p* < 0.01). The dataset of the quantitative PCR experiments is included in Additional file 10: Tab. S2

To analyze the antennal expression of the SNMP1 protein in mutants, immunohistochemical experiments with a polyclonal antibody against the extracellular domain of the WT *S. gregaria* SNMP1 protein were conducted. For this purpose, longitudinal sections through the antenna of male desert locusts were used. Consistent with the results of previous in situ hybridization and immunohistochemical approaches [49–51], immunolabeling was observed around numerous DAPI-stained cell nuclei in WT controls, demonstrating the presence of SNMP1-positive cells (Fig. 2A–F). By contrast, no such immunoreactivity was detectable in the antenna of male SNMP1^−/−^ individuals (Fig. 2G–I). The results of the immunohistochemical experiments were corroborated by Western blot analysis with the antibody against SNMP1. In these immunoblotting experiments with antennal protein fractions from WT or SNMP1^−/−^ locusts, an immunoreactive band of the predicted molecular mass for the SNMP1 protein (~ 57 kDa) was only detectable for the protein fraction from WT animals (Fig. 2J). These findings could be due to the truncation of the SNMP1 protein in SNMP1^−/−^ animals; therefore, it may not be recognized by the antibody used, which targets the extracellular domain of SNMP1. Additionally, SNMP1 expression might be substantially downregulated in SNMP1 mutants. In accordance with this notion, in (non-quantitative) PCR approaches, amplification with an SNMP1-specific primer pair (matching exon 3 and exon 6 of the SNMP1 gene) was obviously weaker using antennal cDNA of mutants as compared to cDNA from the antennae of WT desert locusts (Additional file 3: Fig. S3). Therefore, to assess whether the CRISPR/Cas-induced 22-bp deletion in the SNMP1 gene might affect the expression level of SNMP1-encoding mRNA in desert locusts, quantitative PCR experiments were performed. In these PCR reactions, threshold cycle (CT) values were determined using antennal cDNA from WT and SNMP1^−/−^ animals along with a primer pair matching exon 9 of the SNMP1 gene. In quantitative PCR assays, the housekeeping gene glyceraldehyde-3-phosphate dehydrogenase (GAPDH) was utilized as a reference gene as it has been shown to be useful for normalizing expression in adult *S. gregaria* tissues [53]. Employing the expression of GAPDH as a reference revealed that ∆CT was significantly higher for mutants, indicating a reduced expression of SNMP1 in male and female SNMP1^−/−^ animals (upper panel in Fig. 3). Calculating the relative expression disclosed that expression of SNMP1 mRNA in the antenna is about 96% (males) and 98% (females) lower in mutants than in WT conspecifics (lower panel in Fig. 3). Thus, in summary, the findings of immunohistochemistry, Western blot, and quantitative PCR approaches strongly suggest that SNMP1^−/−^ animals lack functional expression of SNMP1 in their antennae. In this context, and in view of the subsequent physiological and behavioral experiments, we investigated by morphometric analysis whether the lack of functional SNMP1 expression in mutants could affect the development of the antenna that houses the periphery of the olfactory system. For this purpose, we measured the length of the right antenna of adult males of both the WT and the SNMP1^−/−^ strain. Analysis of the antennae from 10 randomly selected individuals of each strain revealed an antenna length of 1.170 cm ± 0.040 cm (mean ± standard error of the mean) for WT animals and 1.210 cm ± 0.023 cm for mutants (Additional file 4: Tab. S1). Since a statistical analysis disclosed no significant differences in antenna length between WT and SNMP1^−/−^ animals, these observations suggest that deletion of functional SNMP1 expression does not generally impair the development of the antenna in mutants.

### SNMP1^−/−^ animals show impaired electrical responses to the courtship inhibition pheromone PAN

**Fig. 4:**
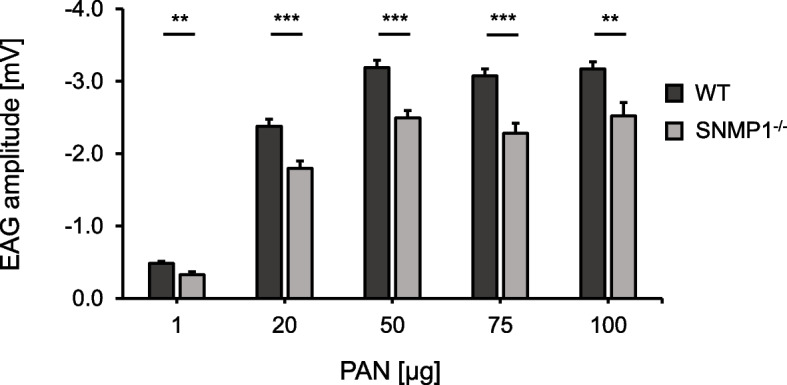
EAG responses to PAN differ significantly between WT and SNMP1^−/−^ male antennae. The bars represent mean EAG amplitudes recorded from the antennae of WT (dark bars) and mutant (light gray bars) males. For each individual, only the right antenna was used. 12 WT and 12 mutant antennae were analyzed for 75 and 100 µg of PAN, while 22 WT and 22 mutant antennae were recorded for 1, 20 µg, and 50 µg of PAN. The standard error of the mean is indicated. Two-tailed *p*-values were determined by unpaired *t*-tests (***, *p* < 0.001; **, 0.001 ≤ *p* < 0.01). The dataset of the EAG recordings with PAN is given in Additional file 11: Tab. S3

Since PAN serves as an important pheromone during courtship in male desert locusts [13], we conducted electroantennography (EAG) recordings with male antennae to scrutinize the relevance of SNMP1 for the peripheral reception of PAN. In antennae from both WT and SNMP1^−/−^ animals, a single puff stimulation with PAN evoked an EAG response characterized by a fast depolarization succeeded by a slower recovery phase leading back to the standing potential (Additional file 5: Fig. S4). Using different amounts of PAN for stimulation (from 1 to 100 µg applied to a small piece of filter paper), it was found that the EAG response amplitude depended on the stimulus intensity, i.e., higher PAN amounts elicited stronger EAG responses (reaching a plateau at 50 µg of PAN; Fig. 4). Importantly, for all amounts of PAN tested, the amplitude of EAG responses was significantly lower in SNMP1^−/−^ than in WT animals (between 32% for 1 µg PAN and 20.5% for 100 µg PAN), indicating reduced antennal responsiveness to PAN in the mutants over a broader range of PAN concentrations (Fig. 4). In EAG experiments with the plant odorant linalool (LIN; 1, 50, or 100 µg of LIN applied to a filter paper), responses were not significantly diminished in SNMP1-deficient males compared to WT conspecifics (Additional file 6: Fig. S5), demonstrating that odorant-induced antennal responses are not generally decreased in mutant animals.

**Fig. 5:**
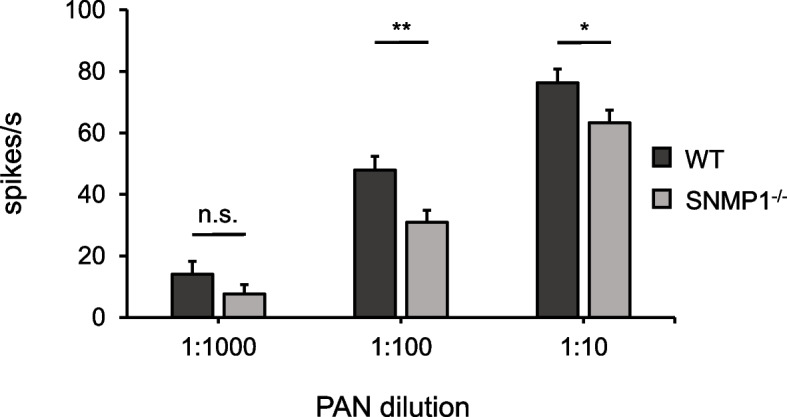
The PAN-induced spiking rate of basiconic sensilla on the antenna is decreased in SNMP1^−/−^ males. PAN-evoked response profiles of basiconic sensilla in the antennae of WT and SNMP1^−/−^ males were examined in SSR experiments using different amounts of PAN. For stimulation, 10 μl of PAN dilution was loaded on a piece of filter paper inserted in a glass Pasteur pipette. The amount of PAN on the filter paper corresponded to 10.2, 102, or 1020 µg for the 1:1000, 1:100, and 1:10 dilution, respectively. The PAN-induced spike rate was calculated as described in detail in the “Methods” section. Dark gray bars represent mean values recorded from 42 basiconic sensilla originating from four WT males. Light gray bars show mean values obtained from 44 basiconic sensilla derived from three SNMP1.^−/−^ males. Error bars denote standard errors of the mean. Two-tailed *p*-values were calculated by unpaired *t*-test (**, 0.001 ≤ *p* < 0.01; *, 0.01 ≤ *p* < 0.05; n.s., no statistical significance). The dataset of the SSR experiments is listed in Additional file 12: Tab. S4

EAG responses are considered to reflect the sum of electrical potentials induced by activated OSNs on the antenna [55, 56]. Among the different olfactory sensilla types in *S. gregaria*, SNMP1 expression seems to be confined to trichoid and basiconic sensilla [48, 49]. Moreover, PAN triggers electrical signals in sensilla basiconica from *S. gregaria*, but not in other sensilla types [15]. Therefore, to examine the relevance of SNMP1 for PAN reactivity in the PAN-sensitive and SNMP1-positive sensilla basiconica in more detail, single sensillum recordings (SSR) were conducted with basiconic sensilla of WT and mutant males (Additional file 7: Fig. S6) because this technique allows (extracellular) recording of action potentials generated by OSNs within a single sensillum [55]. Stimulation of sensilla basiconica from males with different amounts of PAN (10 µl of a 1:10, 1:100, or 1:1000 dilution applied to a filter paper in the stimulus pipette) elicited a dose-dependent increase in spike frequency for both genotypes (Fig. 5). Noteworthy, the spike frequencies of basiconic sensilla were significantly lower in mutants than in WT conspecifics when stimulated with PAN diluted 1:10 or 1:100 (Fig. 5), indicating that SNMP1 is important for PAN-evoked olfactory responses. With a PAN dilution of 1:1000, no significant differences between basiconic sensilla from WT versus SNMP1^−/−^ animals were found (Fig. 5). However, the reduction of PAN-induced reactivity in mutant versus WT animals was higher at a dilution of 1:1000 (decrease of ~ 45%) than at a dilution of 1:100 (~ 36% reduction) or 1:10 (~ 17% decline), suggesting that SNMP1 function might be particularly relevant for PAN detection at lower concentrations. Likewise, also in EAG experiments (Fig. 4), the decrease of PAN-evoked responsiveness observed in the antennae of SNMP1^−/−^ animals was highest (32% reduction) at the lowest amount of PAN tested (1 µg).

### PAN-evoked responses in PNs of the AL are diminished in SNMP1 mutants

**Fig. 6:**
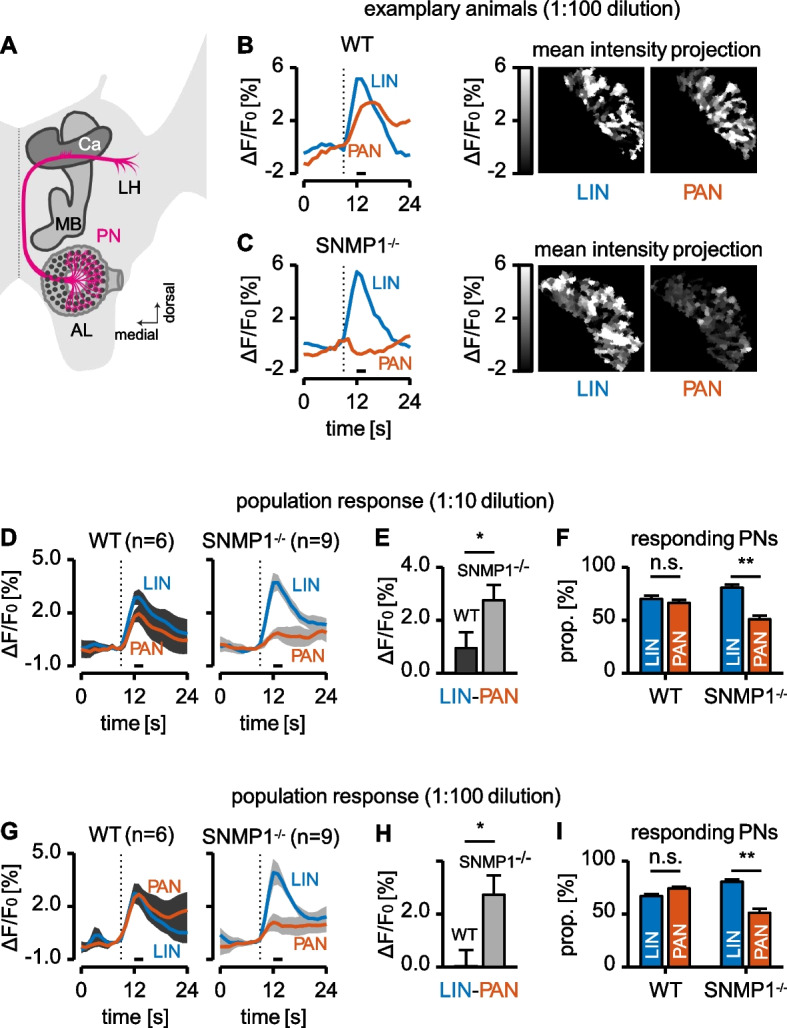
Functional confocal microscopy reveals decreased PAN-induced responses in PNs of SNMP1^−/−^ males. **A** Schematic illustration showing one brain hemisphere (light gray) of a desert locust with a particular emphasis on the antennal lobe (AL) to which antennal OSNs (not shown) send their axons to form glomerular structures (dark dots) with dendrites of projection neurons (PNs; an individual PN is depicted in pink). The PNs convey information to higher-order brain centers, i.e., the lateral horn (LH) and the mushroom body (MB). To selectively backfill PNs in the AL for calcium imaging experiments, a calcium indicator (Cal-520-Dextran) was injected into the calyx (Ca) of the MB. **B**–**I** Calcium responses to PAN or LIN were examined in PNs from the AL of six WT and nine SNMP1^−/−^ males. Odorants were presented to the antenna either as a 1:10 or a 1:100 dilution, and the responses were adjusted by subtracting the responses to the solvent (mineral oil). Activated regions were identified using Otsu’s method on the mean intensity projection (see “Methods” section for details). The dotted vertical lines (**B**, **C**, **D**, and **G**) indicate when the stimulus was applied for 2 s. Horizontal black bars under the time courses (**B**, **C**, **D**, and **G**) represent the time window of analysis (used for mean intensity projections in **B** and **C** and for further analysis in **E**, **F**, **H**, and **I**). *P*-values were determined using a paired two-sided Wilcoxon signed rank test (**D**, **F**, **G**, and **I**) or an unpaired two-sided Wilcoxon rank-sum test (**E** and **H**) (**, 0.001 ≤ *p* < 0.01; *, 0.01 ≤ *p* < 0.05; n.s., no statistical significance). **B,C** Average activity time courses (grand mean across all responding regions) for PAN or LIN (1:100 dilution) based on the results obtained with an exemplary WT (**B**) or mutant (**C**) animal. **D** Average population responses (grand mean across all animals) of WT and mutant males in response to LIN and PAN at a dilution of 1:10. The gray areas indicate the standard error of the mean (*p*-values for the relative change in fluorescence intensity during the time of the analysis window: WT *p* = 0.2188; SNMP1^−/−^ *p* = 0.0039). **E** Difference between the average LIN and PAN responses during the time window of analysis (cf. **D**) for WT and SNMP1^−/−^ animals. **F** Proportion of segmented regions (i.e., PNs) in the AL of WT and SNMP1^−/−^ locusts responding to LIN or PAN, respectively (1:10 dilution). The blue bars indicate the percentage of segmented regions (i.e., PNs) activated by LIN out of all segmented regions in the AL of an animal. Likewise, the orange bars denote the percentage of PAN-responding PNs out of all PNs. Remarkably, some PNs were observed to respond to LIN and PAN (therefore, for each genotype, the sum of the percentages from both bars is higher than 100%). **G–I** The same experiments as in **D**–**F**, but with 1:100 dilutions of PAN and LIN. The *p*-values in **G** for the relative change in fluorescence intensity during the analysis window are: WT p > 0.99; SNMP1^−/^.^−^ *p* = 0.0078. The datasets of the calcium imaging experiments are given in Additional file 13: Tab. S5

While the EAG and SSR experiments demonstrated reduced electrical reactivity to PAN in antennal OSNs of SNMP1-deficient locusts, it is unclear whether this decrease of PAN-induced electrical signals in OSNs affects neuronal activity in the brain. To address this issue, we monitored the activity of PNs in the AL, the first relay station for olfactory information in the brain. Similar to previous studies in other insects [57, 58], functional calcium imaging was used to simultaneously inspect odorant-induced activity in large numbers of PNs across the AL. Therefore, PNs in the AL of male WT and SNMP1^−/−^ desert locusts were selectively backfilled with a calcium-sensitive dye by injecting the dye into the calyx of the mushroom bodies (Fig. 6A) before stimulating the antenna with PAN or the plant odorant LIN (in 1:10 and 1:100 dilutions). Subsequent monitoring of calcium dynamics in the AL from individual animals enabled the assessment of odorant-induced activation of the AL (Fig. 6B–C). The data obtained by quantifying responses to PAN and LIN in the same AL preparation demonstrated obvious differences between WT and mutant animals; these differences were observed for both odorant dilutions (Fig. 6D and G). In WT animals, both exposure to PAN and exposure to LIN elicited intense calcium signals in the AL. It is noteworthy that in WT animals, there was no substantial difference in the intensity of AL responses to LIN and PAN. For SNMP1^−/−^ conspecifics, however, activation of the AL upon exposure to LIN was significantly stronger than following exposure to PAN (Fig. 6D and G). Consequently, the difference between the magnitude of the LIN and PAN response was significantly lower in WT than in SNMP1^−/−^ animals (Fig. 6E and H). To investigate whether a reduced number of activated PNs may account for the decreased PAN-evoked responses in the AL of mutants, the proportion of responding PNs (i.e., the proportion of responding regions based on Otsu’s thresholding method) was determined. These analyses revealed that in WT males, the proportion of PNs responding to PAN or LIN was similar; this observation was made for both dilutions (Fig. 6F and I). In mutant males, by contrast, for both dilutions, the proportion of PNs activated by PAN was significantly lower than that for LIN (Fig. 6F and I). In summary, the findings of the calcium imaging experiments demonstrate that the elimination of functional SNMP1 not only interferes with PAN detection in the periphery, but in consequence also impairs PAN-induced responses in brain regions processing the incoming olfactory information.

### PAN-controlled behavior is hampered in SNMP1 mutants

**Fig. 7:**
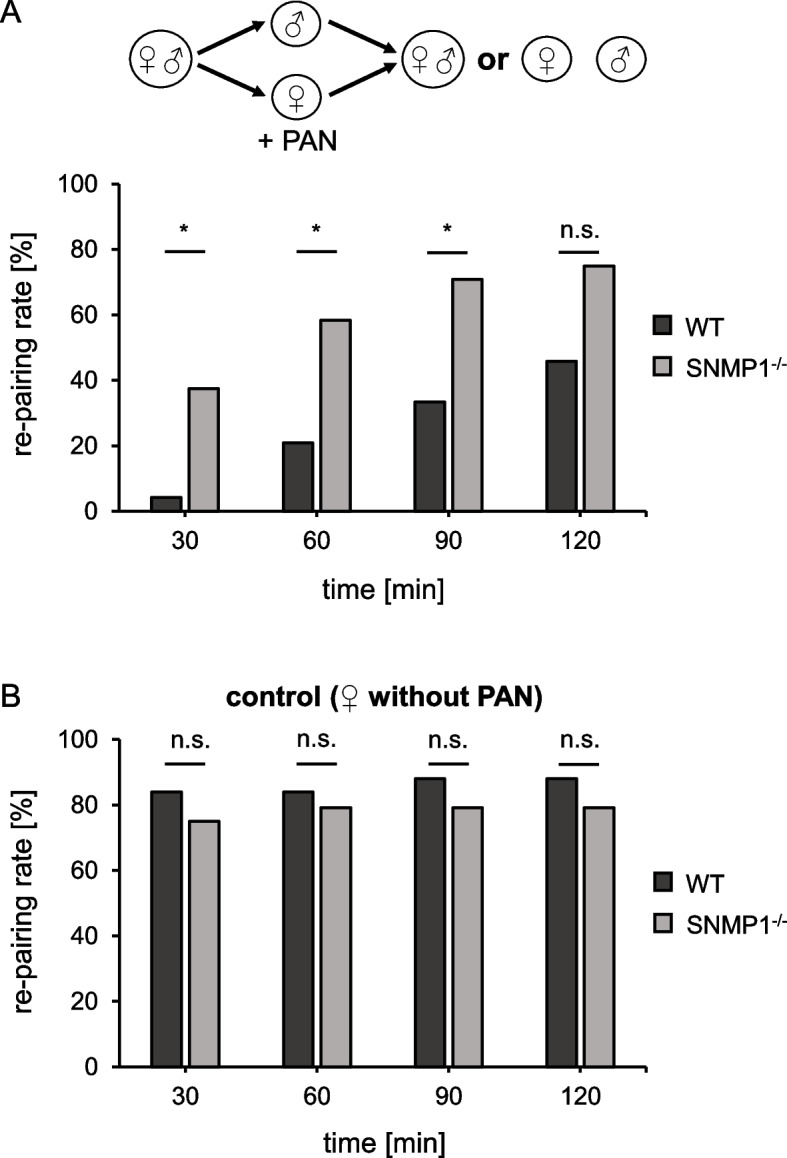
Mutation of SNMP1 impairs PAN-affected re-pairing behavior of desert locusts. **A** Re-pairing rate of WT and mutant desert locusts painted with PAN. The female of a pair of WT or SNMP1^−/−^ animals (placed individually in a separate cage) was briefly taken out of the cage, painted with 1 µl of 10% PAN, and placed back in the cage. Re-pairing with the male was monitored for 2 h in 30-min intervals. For each time point, the re-pairing rate was calculated as 100% × the number of re-paired couples divided by the total number of couples. The data depicted were derived from 24 couples for each genotype. **B** Same experiment as in A, but without painting of females with PAN. The data shown are based on 25 WT and 24 SNMP1^−/^.^−^ couples. The *p*-values (both tails) were determined by Fisher’s exact test (*, 0.01 ≤ *p* < 0.05; n.s., no statistical significance). The dataset of the re-pairing assays with and without PAN are shown in Additional file 14: Tab. S6

To assess whether the reduced responses to PAN in SNMP1 mutants also affect PAN-elicited behavior(s), we adopted two behavioral approaches that had unraveled the role of PAN as a male-released courtship inhibition pheromone [13]. In the first experimental setting designated as re-pairing assay, a pair of either WT or SNMP1^−/−^ animals was placed in a small cage. Subsequently, the female was taken out of the cage, and its pronotum was painted with 1 µl of 10% PAN diluted in the solvent dichloromethane (CH_2_Cl_2_). The female was then returned to the cage with the male, and re-pairing was monitored in 30-min intervals for 2 h. In this approach, the percentage of re-pairing was comparatively low in the early phase of the experiment (after 30 min), but increased over time for both genotypes (Fig. 7A), presumably due to evaporation of PAN. Strikingly, at all time points examined, the rate of re-pairing was clearly lower in WT than in SNMP1^−/−^ couples; for 30, 60, and 90 min after the onset of the observation period, the difference in re-pairing between the two genotypes was statistically significant (Fig. 7A). This observation indicates a reduced (behavioral) responsiveness to PAN in SNMP1^−/−^ animals. Yet, it cannot be ruled out that the re-pairing rate is generally different between WT and SNMP1^−/−^ couples. Therefore, control experiments were conducted with WT and SNMP1^−/−^ couples treated as described above but lacking painting with PAN. In these approaches, the rate of re-pairing was high for both genotypes, ranging from 75 to 88%. For all time points tested, no significant difference was found between WT and SNMP1^−/−^ couples (Fig. 7B), indicating that both genotypes re-pair at a similar level. To assess a possible effect of the solvent CH_2_Cl_2_, further control experiments were conducted with only WT couples in which females were either painted with 1 µl CH_2_Cl_2_ or left unpainted. In this approach, no significant differences in re-pairing were observed between pairs with CH_2_Cl_2_-treated females and pairs with unpainted females (Additional file 8: Fig. S7). As CH_2_Cl_2_ does not prevent re-pairing in WT animals, this finding suggests that the observed lower re-pairing rate in WT versus SNMP1^−/−^ couples (Fig. 7A) can be traced to PAN. Thus, SNMP1^−/−^ male locusts seem to be severely impaired concerning proper responses to PAN in a behavioral experiment exploring re-pairing.

**Fig. 8:**
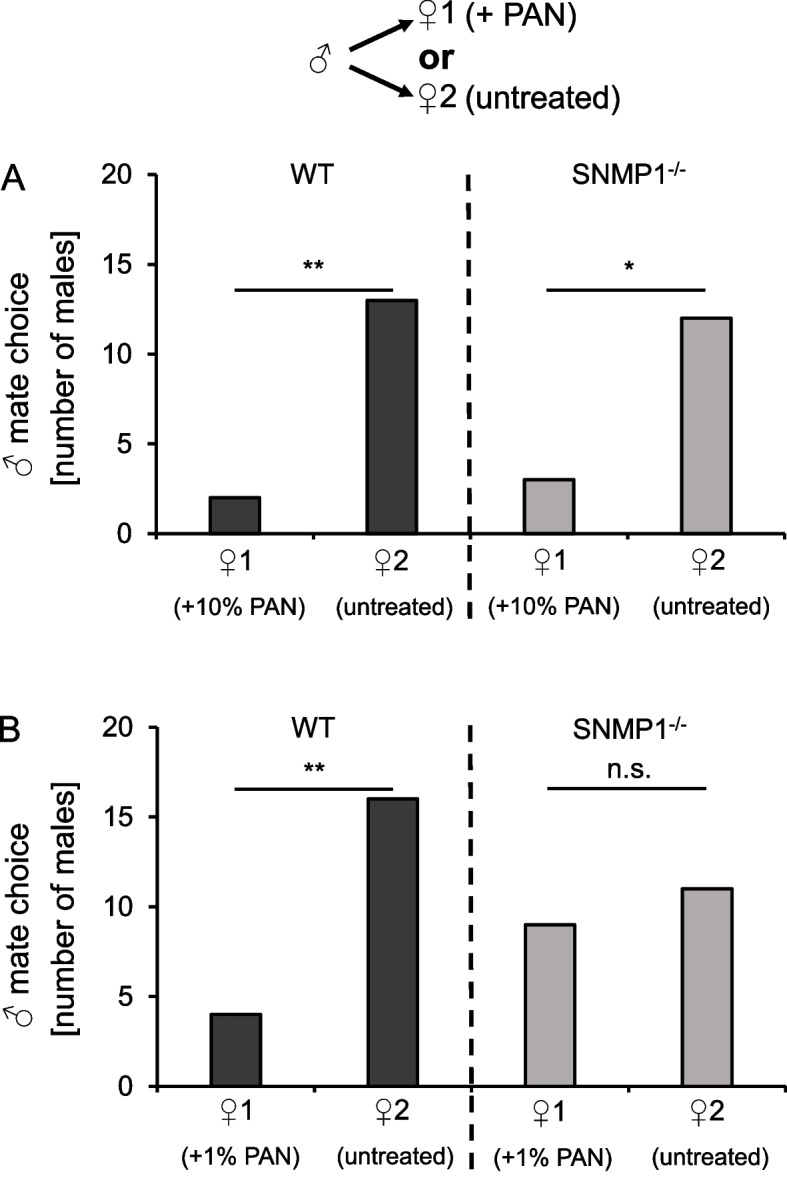
Low concentrations of PAN affect mate choice in WT but not in SNMP1^−/−^ males. **A** In a cage with a pair of WT or SNMP1^−/−^ animals, a second female (♀2) from another pair of the same genotype was added. The original female (♀1) was treated with 1 µl of 10% PAN, whereas the second female (♀2) was not painted with this substance. Within a 30-min interval, it was observed with which female the male pairs first. For each genotype, 15 males were tested. In both WT and mutant animals, the males significantly preferred the untreated female (♀2) to the PAN-painted one (♀1). **B** Same experiment as in **A**; but this time, ♀1 was treated with 1 µl of only 1% PAN. Twenty males were used for each genotype. In WT locusts, the males significantly preferred the untreated female (♀2), whereas SNMP1^−/^.^−^ males showed no preference (*p* = 0.655). *P*-values were calculated using a chi-square goodness of fit test (**, 0.001 ≤ *p* < 0.01; *, 0.01 ≤ *p* < 0.05; n.s., no statistical significance). The dataset of the mate choice assays are included in Additional file 15: Tab. S7

In a second experimental set-up, the so-called mate choice assay, *S. gregaria* WT and SNMP1^−/−^ males were given the choice between two females of the corresponding genotype. One female (♀1) was painted with PAN (1 µl of either a 1% or a 10% dilution in CH_2_Cl_2_), the other (♀2) remained untreated. In experiments with females treated with 10% PAN (♀1), males from both genotypes preferentially paired with untreated females (♀2) (Fig. 8A). A similar preference was observed in WT males when choosing between a female painted with 1% PAN (♀1) and an untreated one (♀2) (Fig. 8B). By contrast, mutant males showed no significant preference for untreated females over those painted with 1% PAN (Fig. 8B), indicating that SNMP1^−/−^ males were impaired in detecting PAN appropriately at lower concentrations. Taken together, the findings of the re-pairing and mate choice assays strongly suggest that in *S. gregaria*, SNMP1 plays a role for adequate behavioral responses to PAN.

## Discussion

Here, we show that the sensory neuron membrane protein 1 (SNMP1) is crucial for pheromone detection in the desert locust. Through CRISPR/Cas gene editing and a series of physiological and behavioral experiments, we demonstrate that SNMP1 deficiency substantially diminishes locusts’ responses to the courtship inhibition pheromone phenylacetonitrile (PAN), altering PAN-induced behaviors that are associated with reproduction.

In OSNs of holometabolous insects, such as flies and moths, SNMP1 is critical for detecting pheromonal compounds comprising long carbon chains [35, 37–39]. By contrast, the role of SNMP1 in the olfactory system of hemimetabolous insect species is elusive. SNMP1 appears to be of particular importance in the hemimetabolous desert locust, as it is co-expressed with a considerable number of OR types [50, 51], suggesting that SNMP1 might contribute to detecting pheromonal compounds with different molecular structures. To date, our knowledge about pheromones in *S. gregaria* is limited, and pheromonal substances with long carbon chains have not been described in this species. One of the best-studied pheromones in the desert locust is PAN, an aromatic compound serving as a courtship inhibition pheromone [12–14]. To investigate the role of SNMP1 in locust pheromone detection, we analyzed its relevance for sensing PAN through physiological and behavioral experiments. For this purpose, we set out to generate an SNMP1-deficient mutant line by CRISPR/Cas-mediated gene editing in *S. gregaria*. CRISPR/Cas-based methods were previously utilized to edit the genome of different insect species [59, 60]. However, to our knowledge, CRISPR/Cas-mediated genetic manipulation has not yet been successfully applied to the desert locust. Given the destructive impact of *S. gregaria* on agriculture and global food security, targeting genes that govern reproductive processes offers a promising avenue for a purposive control of outbreaks [61]. In this regard, CRISPR/Cas-based editing of olfactory genes contributing to the detection of pheromones such as PAN, which are associated with courtship or other reproductive behaviors, could be of special interest. Here, we generated a mutant line carrying a 22-bp deletion in exon 4 of the SNMP1 gene, leading to a frameshift and a massively truncated protein (Fig. 1 and Additional file 2: Fig. S2). Verifying the 22-nucleotide deletion in the mRNA coding for SNMP1, we observed a dramatic reduction of the mRNA expression level for SNMP1 in the antenna of mutant animals (Fig. 3). This result is in line with a previous finding in the related locust species *Locusta migratoria*, in which a CRISPR/Cas-based mutation of the odorant receptor co-receptor ORCO led to a significant decrease in the ORCO-encoding mRNA level in antennal tissue [62]. Although the precise processes underlying such strongly reduced mRNA expression in mutants are not completely understood, it has to be considered that Cas9-mediated cleavage of genomic DNA generally evokes a double-strand break that activates DNA damage repair mechanisms, including the non-homologous end joining pathway. Repair via this pathway usually causes deletions and/or insertions close to the site of the double-strand break that frequently induce a frameshift, leading to a premature termination codon [63]. Subsequently, RNA transcripts that contain a premature termination codon resulting in a truncated coding sequence are recognized by the nonsense-mediated mRNA decay pathway that degrades such RNA molecules [64]. Accordingly, the massive reduction in the expression of SNMP1-encoding mRNA in the SNMP1^−/−^ line (Fig. 3) could be attributed to nonsense-mediated mRNA decay of this transcript. In summary, due to the truncated variant of the SNMP1 protein and the severely impaired expression level of the corresponding mRNA, individuals of the SNMP1^−/−^ line seem to lack a functional SNMP1 protein. The absence of functional SNMP1 was validated by immunohistochemical staining approaches with an antibody targeting the extracellular domain of SNMP1, in which we could not observe labeling in antennal sections from mutants (Fig. 2).

The notion that SNMP1 is vital for pheromone detection in hemimetabolous locusts — similar to holometabolous flies and moths — was corroborated by electrophysiological EAG (Fig. 4) and SSR (Fig. 5) experiments. In these approaches, PAN-induced responses were significantly lower in mutant animals compared to WT conspecifics, indicating that SNMP1 is important for detecting PAN, notably for the strength of the PAN-evoked electrical reactivity. It is noteworthy that the reduction of PAN-elicited responses in mutants was highest at the lowest PAN concentrations tested, suggesting that SNMP1 plays a critical role in the sensitivity of PAN detection. However, as EAG and SSR responses to PAN were still detectable in SNMP1^−/−^ animals (Figs. 4 and 5), SNMP1 does not appear to be required per se for the responsiveness of antennal OSNs to PAN. This concept is also consistent with a significant reduction — but not absence — of PAN-induced activity in PNs of the AL of mutants (Fig. 6). At first glance, these findings seem to contradict earlier observations in *Drosophila melanogaster*, in which responses to the pheromone cVA in OSNs from T1 sensilla were completely abolished in SNMP1-deficient mutants [35–37, 65]. In these OSNs from T1 sensilla of mutant flies, only upon application of nonphysiologically high cVA doses, (weak) responses were observable [65]. Similar to *Drosophila melanogaster*, in the moth *Helicoverpa armigera*, so-called B-neurons in type C trichoid sensilla almost completely lost responsiveness to certain pheromonal ligands upon elimination of SNMP1 [38]. Consequently, in given OSNs from *Drosophila melanogaster* and *Helicoverpa armigera*, SNMP1 seems to be indispensable for pheromone-evoked responses. In this context, it remains uncertain why a clear reactivity to the pheromone PAN is retained in SNMP1^−/−^ individuals from *S. gregaria*. Since the processes underlying the residual reactivity to PAN in SNMP1-deficient desert locusts are unknown, one possible explanation might be that PAN activates not only SNMP1-positive but also (subsets of) SNMP1-negative OSNs in WT *S. gregaria*; the latter OSN population would most likely preserve its responsiveness to PAN in SNMP1 mutants. Alternatively, PAN-induced responses in locust OSNs that express SNMP1 in WT animals could be only reduced but not abolished following the deletion of SNMP1. Such a scenario would be reminiscent of the A-neurons in type A trichoid sensilla of *Helicoverpa armigera* that are still responsive to their cognate pheromonal ligand in SNMP1 mutants, albeit with a lower sensitivity, i.e., higher concentrations of the ligand are required to elicit a detectable response. Moreover, the pheromone-induced responses in these neurons are significantly lower in SNMP1-deficient moths than in WT conspecifics, even when high concentrations of the pheromonal compound are applied [38]. Thus, in A-neurons from type A trichoid sensilla of *Helicoverpa armigera*, SNMP1 is essential for the sensitivity of pheromone-evoked reactivity, but not indispensable for responsiveness to pheromones per se. Such a response pattern to pheromonal compounds would be consistent with the results of the mate choice experiments of the present study. In these behavioral approaches, compared to WT animals, a higher concentration of PAN was required to elicit an appropriate reaction in mutant male locusts, i.e., preference of untreated over PAN-treated females (Fig. 8). Taken together, the results of the electrophysiological recordings, the calcium imaging and the behavioral experiments demonstrate that SNMP1 substantially contributes to the detection of PAN. Thus, SNMP1 is involved in pheromone sensing in the hemimetabolous species *S. gregaria* — similar to holometabolous insects [35, 37–39].

In contrast to the pheromones of flies and moths, whose detection relies on SNMP1, PAN is not a long-chain lipid, but an aromatic compound without long carbon chains. Consequently, in the desert locust, for which no long-chain pheromones have been described to date, SNMP1 seems to facilitate the sensitive detection of pheromones with other molecular traits, including aromatic substances such as PAN. Whether SNMP1 might contribute to detecting aromatic compounds in other species, most notably holometabolous insects, remains unclear. So far, in *Helicoverpa armigera*, testing of some aromatic chemicals (including methyl benzoate, methyl salicylate, and salicylaldehyde) revealed that their reception is not affected in the antenna of SNMP1-deficient moths [38]. Thus, SNMP1 types of holometabolous flies and moths might mainly contribute to the detection of long-chain aliphatic pheromones [35, 37, 38], whereas SNMP1 of the hemimetabolous desert locust is involved in the reception of aromatic compounds (this study). In this context, the question arises whether SNMP1 types from different insect orders, e.g., dipterans (flies), lepidopterans (moths), and orthopterans (locusts), can be categorized into subgroups according to their relevance for the detection of certain classes of chemicals and whether such a subgrouping might be reflected in the relatedness of the SNMP1 amino acid sequences. According to this notion, SNMP1 types from dipterans and lepidopterans that both use SNMP1 for receiving long-chain aliphatic chemicals [35, 37, 38] might be more closely related to each other than to SNMP1 from the desert locust, in which SNMP1 is critical for detecting an aromatic compound. Comparing the SNMP1 amino acid sequence of *S. gregaria* with that of *Drosophila melanogaster* and *Helicoverpa armigera* revealed ~ 37 and ~ 40% identity, respectively, while the SNMP1 types of the latter two species are ~ 39% identical. Therefore, based on these rather moderate and similar percentages of amino acid sequence identities of SNMP1 from these three orders, it is difficult to predict subgroups of SNMP1 types critical for the detection of alike or totally different chemicals. In this regard, it is noteworthy that OSNs of *Drosophila melanogaster* co-expressing the OR type OR19a and SNMP1 respond to limonene [1-methyl-4-(1-methylethenyl)cyclohexene] [35, 66–68], a plant odorant that is not an aliphatic substance with a long carbon chain, but belongs to the group of cyclic monoterpenes. Although limonene is not an aromatic compound, like PAN, it comprises a ring of six carbon atoms and relatively short side chains. Whether SNMP1 contributes to the detection of limonene in the OR19a-positive OSNs is unknown. However, similar to the fly pheromone cVA, limonene binds to the SNMP1-related mammalian protein CD36 that can replace SNMP1 function in OSNs of *Drosophila melanogaster* [36]. Thus, the relevance of SNMP1 for detecting aromatic/cyclic compounds might not be confined to hemimetabolous locusts. Moreover, in *Drosophila* flies, SNMP1 could mediate the detection of very different chemicals, including the long-chain aliphatic pheromone cVA and the cyclic monoterpene limonene. In this regard, it is important to emphasize that the ligand specificity of an OSN is ultimately determined by the olfactory receptor type expressed. Consequently, it is conceivable that SNMP1 in a given insect species enables the detection of a wider range of substances, while the co-expressed ORs are more narrowly tuned to ligands from a particular class of chemicals. In such a scenario, SNMP1 would “only” facilitate the access of a ligand to the ligand-binding site of a cognate OR, while the chemical nature of the ligand itself might be less important in relation to SNMP1. This concept is consistent with previous findings on SNMP1 in *Drosophila* flies suggesting that it acts as a funnel that transports the pheromone cVA via its large tunnel-like ectodomain to the specific receptor OR67d [36]. The observation that 33 OR types were found co-expressed with SNMP1 in *S. gregaria* supports the notion that SNMP1 could contribute to the detection of a broader spectrum of odorants [51]. However, the ligands of the OR types of *S. gregaria* that are co-expressed with SNMP1 have not yet been identified, limiting our current knowledge of odorants whose proper recognition depends on SNMP1. Given that only a subpopulation of OR types is co-expressed with SNMP1 in flies, moths, and locusts, the question arises as to why some OR types require SNMP1 for efficient ligand delivery to the receptor while others do not. Whether this is due to the chemical properties of the respective odorants or depends on specific structural characteristics of the relevant OR types remains the subject of future investigations. Therefore, more comprehensive functional studies are needed to scrutinize the role of SNMP1 in the detection of aromatic and other compounds across different hemimetabolous and holometabolous insects.

## Conclusions

This study demonstrates a critical role of SNMP1 in the sensitive recognition of an aromatic pheromone compound, suggesting that the function of SNMP1 extends far beyond its previously reported requirement for the detection of long-chain aliphatic pheromones. Moreover, our findings not only highlight for the first time the crucial importance of SNMP1 for proper chemical communication and reproductive behavior in a hemimetabolous insect pest, but may also suggest SNMP1 as a potential target for future alternative locust control strategies.

## Methods

### Animals

Gregarious desert locusts, *Schistocerca gregaria* (Forskal), were reared under crowded conditions as described previously [14, 49]. In brief, the animals were kept in metal cages (50 × 50 × 50 cm) with a light cycle of 12L:12D. The temperature was 34 °C during the day and 27 °C at night. The locusts were fed with fresh wheat seedlings and oat flakes.

### Design of guide RNA

Employing a draft genome of *S. gregaria* [69] available via ORCAE (http://bioinformatics.psb.ugent.be/blast/moderated/?project=orcae_Schgr), the nucleotide sequence encoding the *S. gregaria* SNMP1 protein (GenBank/NCBI: KU659599.1 and XM_049985376.1) was used as a query for a BLAST analysis (program: blastn; database: Schgr_genome). This bioinformatic approach revealed that the coding sequence of *S. gregaria* SNMP1 is derived from nine exons in total. A fragment of 20 nucleotides (5′-GTGCGCAACTTCCTCTTCGA) situated directly upstream of a (potential) PAM site (5′-CGG) in exon 4 was chosen as guide RNA (Additional file 1: Fig. S1). This selected sequence motif was submitted to the Sigma-Aldrich/Merck custom CRISPR service (Merck, Darmstadt, Germany) for synthesis of a single-guide RNA that includes both the guide RNA and a Cas nuclease-recruiting sequence (tracrRNA); the latter sequence was provided by the manufacturer. With the guide RNA sequence used for CRISPR/Cas-mediated generation of mutants, we screened the genome of *S. gregaria* for potential off-target sites utilizing the Cas-OFFinder tool (http://www.rgenome.net/cas-offinder/), an algorithm for identifying off-target sites of Cas9 RNA-guided endonucleases [70]. With this program, no off-target sites were found in the reference genome of *S. gregaria* (submitted GenBank assembly/NCBI: GCA_023897955.2) when no or only one mismatch was allowed. Considering up to three mismatches, three possible off-target sites were detected in the genome of the desert locust. Importantly, they were all located outside of annotated genes.

### Generation of mutant locusts

Adult male and female desert locusts were kept together, and the females were allowed to lay their eggs in plastic cups (about 10 cm high) filled with humid sand. When locusts were observed to lay eggs, the eggs were immediately taken out of the sand and cleaned with Ringer solution (9.82 g/l NaCl; 0.48 g/l KCl; 0.19 g/l NaH_2_PO_4_; 0.25 g/l NaHCO_3_; 0.73 g/l MgCl_2_; 0.32 g/l CaCl_2_; pH 6.5). Eggs were isolated and placed on a filter paper (MN 640 we ashless; Macherey–Nagel, Düren, Germany) covering an agarose plate [1% agarose (Genaxxon Bioscience, Ulm, Germany) dissolved in deionized water]. Next, lyophilized Cas9 enzyme (Sigma-Aldrich, St. Louis, MO, USA) was reconstituted as recommended by the manufacturer and diluted with the supplied dilution buffer to a final concentration of 400 ng/µl. The single-guide RNA was reconstituted and diluted in deionized H_2_O to achieve a final concentration of 150 ng/µl. Subsequently, 50 µl of the Cas9 solution was mixed with 50 µl of the single-guide RNA-containing solution before this mixture was injected into eggs (not later than 30 min after laying) using a Femtojet electronic microinjector (Eppendorf, Hamburg, Germany) and glass capillaries. For preparing the capillaries, capillary tubes (3–000-203-G/X, 1.14 mm outer diameter × 3.5 inches length; Drummond Scientific, Broomall, PA, USA) and a horizontal Pul-1 micropipette puller (World Precision Instruments, Sarasota, FL, USA) were utilized (settings: 4 for “delay” and 9 for “heat”). For injection, the capillary was filled with a few microliters of the solution containing Cas9 and the single-guide RNA. Injection was carried out for 0.1 s with an injection pressure (pi) of 600–700 hPa and a compensation pressure (pc) of 60–70 hPa. Subsequently, injected eggs were placed on a filter paper covering a 1% agarose plate. The plate was covered with a lid and kept in an incubator at 30 °C until hatching. Approximately 14 days after injection, nymphs hatched from the eggs and grew into adults (generation G0).

To screen for mutations in exon 4 of the SNMP1 gene, genomic DNA was prepared from the distal part of the wings of adults of generation G0. The tissue was cut into small pieces and incubated for 30 min at 95 °C and 350 rpm in 45 µl of 50 mM NaOH using a thermomixer (Eppendorf). After adding 5 µl of 1 M Tris–HCl (pH 8.5), the supernatant containing the genomic DNA was transferred into a new reaction tube and stored at 4 °C. For subsequent PCR amplification, a forward primer (5′-CTGCTGACCCTGGAGCGCGACAAG) and a reverse primer (5′-TCGCCCTCGCGCTTGAGCTCCTTG) were utilized, matching to the 5′ and the 3′ end of exon 4 of the SNMP1 gene, respectively (all oligonucleotide primers used in this study for PCR amplification were ordered from Biomers.net, Ulm, Germany). PCR reactions were prepared using 39.5 μl H_2_O, 5 μl 10 × Titanium Taq PCR Buffer (Takara Bio, Saint-Germain-en-Laye, France), 1 μl 10 mM 2'-deoxynucleoside 5'-triphosphate (dNTP) solution mix (New England Biolabs, Ipswich, MA, USA), 0.5 μl 100 μM forward primer, 0.5 μl 100 μM reverse primer, 3 μl genomic DNA, and 0.5 μl 50 × Titanium Taq DNA polymerase (Takara Bio). Thermocycling conditions were 1 min at 97 °C succeeded by 35 cycles at 97 °C for 30 s and 3 min at 68 °C. The final cycle was followed by a 3-min incubation at 68 °C. Next, PCR products were visualized using agarose gel electrophoresis and ethidium bromide staining. Amplicons with the expected size of ~ 200 bp were purified using the Monarch DNA gel extraction kit according to the instructions of the manufacturer (New England Biolabs) and sent to Microsynth (Balgach, Switzerland) for Sanger sequencing. If sequencing led to an electropherogram not identical to WT controls (Fig. 1A), the relevant animals of the G0 generation were considered potential mutants for SNMP1 and were allowed to mate individually with a WT conspecific of the opposite sex. Animals of the resulting G1 generation were analyzed for mutations as described above. Purified PCR products from the G1 generation were cloned into the pGEM-T easy plasmid (Promega, Madison, WI, USA) and subjected to sequencing and detailed sequence analysis. Heterozygous G1 individuals carrying a given 22-bp deletion in exon 4 of the SNMP1 gene (Fig. 1B) were mated with each other to obtain a G2 generation. Animals of the G2 generation homozygous for the above-described 22-bp deletion were identified by PCR with genomic DNA and the aforementioned primers followed by direct sequencing of the PCR product; such individuals were used to establish the homozygous SNMP1^−/−^ line.

### Immunohistochemistry

With a few modifications, immunohistochemical experiments were conducted as described recently [49, 71]. Antennae were surgically removed from adult male *S. gregaria* of the WT and the SNMP1^−/−^ line and frozen onto a specimen chuck at − 57 °C in Tissue-Tek O.C.T. Compound (Sakura Finetek, Alphen aan den Rijn, The Netherlands) on the cryobar of a Cryostar NX50 cryostat (Thermo Fisher Scientific, Waltham, MA, USA). Longitudinal antennal sections (12 μm thick) were prepared with the cryostat at a chamber temperature of − 25 °C (temperature of the object head: − 10 °C). Antennal sections were immediately thaw-mounted on Epredia SuperFrost Plus adhesion slides (Thermo Fisher Scientific) and stored at − 25 °C until they were encircled with a colorless ROTI Liquid Barrier Marker (Carl Roth, Karlsruhe, Germany). For fixation, sections were incubated in 4% paraformaldehyde dissolved in PBS (0.85% NaCl; 1.4 mM KH_2_PO_4_; 8 mM Na_2_HPO_4_; pH 7.4) supplemented with 0.1% Triton X-100 at 4 °C for 22 min. Next, sections were washed at room temperature twice in PBS for 5 min and in PBS supplemented with 0.01% Tween 20 for 5 min. Subsequently, sections were rinsed in 50 mM NH_4_Cl (dissolved in PBS) for 5 min and in PBS for 5 min. Following a 30-min incubation at room temperature in blocking solution (10% normal goat serum and 0.5% Triton-X100 in PBS), the sections were incubated overnight at 4 °C (in a humid box) with a primary antibody raised in rabbit against the extracellular domain of SNMP1 from *S. gregaria* [49]. For this incubation, the antibody against SNMP1 was diluted 1:500 in blocking solution. After washing the slides three times for 5 min with PBS, secondary detection was carried out for 1 h at room temperature (in a humid box) with a goat anti-rabbit AF488-conjugated secondary antibody (Jackson ImmunoResearch, Ely, United Kingdom) diluted 1:1000 in PBS. The solution containing the secondary antibody was supplemented with 2 µg/ml of the nuclear stain DAPI (4′,6-diamidino-2-phenylindole). Finally, the slides were washed three times for 5 min in PBS and then mounted in Mowiol solution (20 g Mowiol 4–88, 80 ml PBS, 40 ml glycerol, 2.4 g propylgallat).

Immunohistochemical staining experiments were analyzed with a Leica DMi8 microscope (Leica Microsystems, Wetzlar, Germany). Images were processed using the LAS X software (Leica Microsystems).

### Western blot

Antennae of WT and SNMP1^−/−^ adults were frozen in liquid nitrogen and homogenized using a mortar and a pestle cooled down with liquid nitrogen. The homogenates were mixed in fresh reaction tubes with four times the volume of lysis buffer. Prior to use, 100 ml of lysis buffer (50 mM Tris–HCl pH 7.4; 150 mM NaCl; 1 mM EDTA; 1% Triton X-100; 5% glycerol) was supplemented with one tablet of SigmaFAST protease inhibitor cocktail (Merck). The samples were then incubated on an overhead shaker at 4 °C for 5 h, followed by centrifugation at 5000* g* and 4 °C for 10 min. The supernatant was used for sodium dodecyl sulfate–polyacrylamide gel electrophoresis (SDS-PAGE) and subsequent Western blot analysis. To this end, an aliquot containing 10 µg (or 5 or 15 µg) of the extracted total protein was mixed with an appropriate volume of 5 × sample buffer (0.3 M Tris pH 6.8; 50% glycerol; 0.02% bromophenol blue; 2.5% SDS; 10% β-mercaptoethanol), heated for 5 min at 90 °C and loaded on a lane of a 12% polyacrylamide gel (supplemented with 0.1% SDS). Electrophoresis of two identically loaded gels was run in parallel before one of them was stained with Coomassie blue (upper panel in Additional file 9: Fig. S8), while the proteins in the other gel were electroblotted for 1 h at 200 mA onto a methanol-treated PVDF membrane (Carl Roth) soaked in transfer buffer (25 mM Tris; 192 mM glycine; 20% methanol) using a semi-dry blotting device. Subsequently, the membrane was treated for 1 h with 7% milk powder in TBST (100 mM Tris; 150 mM NaCl; pH 7.5; 0.05% Tween 20) prior to overnight incubation at 4 °C in the SNMP1 antibody diluted 1:1000 in TBST supplemented with 3.5% milk powder. After washing the membrane three times for 10 min in TBST, it was incubated for 1 h at room temperature in alkaline phosphatase-conjugated goat anti-rabbit antibody (Thermo Fisher Scientific) diluted 1:10,000 in TBST with 3.5% milk powder. Next, the membrane was washed three times for 10 min in TBST and twice for 10 min in substrate buffer (100 mM Tris–HCl pH 9.5; 100 mM NaCl; 5 mM MgCl_2_). Finally, the membrane was incubated for 2 h in substrate buffer containing 25 µg/ml nitro blue tetrazolium (NBT) and 50 µg/ml 5-brom-4-chlor-3-indolyl phosphate (BCIP) (lower panel in Additional file 9: Fig. S8). The color reaction was stopped by a 5-min incubation in H_2_O.

### RNA isolation and cDNA synthesis

Twenty antennae from adult (female or male) WT or SNMP1^−/−^ locusts were dissected and immediately frozen in liquid nitrogen. The tissue was homogenized on ice in 1 ml Trizol reagent (Thermo Fisher Scientific) for 8 min with a Dounce homogenizer, a Teflon pestle, and a stirring unit. Extraction of total RNA using Trizol reagent was conducted according to the manufacturer’s protocol. Total RNA was finally resuspended in 20 µl H_2_O, and the concentration of RNA was determined using an Epoch microplate spectrophotometer (BioTek, Winooski, VT, USA). To eliminate contaminating genomic DNA, 18 µl of the RNA-containing solution were mixed with 2 µl of 10 × DNase I reaction buffer and 1 µl of RNase-free DNase I (New England Biolabs) and incubated for 30 min at 37 °C.

For the isolation of poly(A)^+^ RNA from total RNA, magnetic Dynabeads coupled with Oligo(dT)_25_ (Thermo Fisher Scientific) were used in conjunction with a MagneSphere magnetic separation stand (Promega). Before extracting poly(A)^+^ RNA with Dynabeads, the beads were washed. To this end, depending on the amount of total RNA, a given amount of Dynabeads were washed as recommended by the supplier (Thermo Fisher Scientific): for 75 µg of total RNA, 100 µl of Dynabeads was used for washing. Finally, the washed beads were resuspended in a total of 20 µl of binding buffer (20 mM Tris–HCl pH 7.5; 1 M LiCl; 2 mM EDTA). The 21 µl of total RNA treated with DNAse I were heated to 65 °C for 2 min and placed on ice immediately. Next, 20 µl of washed and resuspended Dynabeads was added before the mixture was rotated for 5 min at room temperature. The tube was placed on the magnetic stand for 30 s, and the supernatant was transferred to a novel tube (tube A) on ice. Dynabeads with bound poly(A)^+^ RNA were washed twice using wash buffer B (10 mM Tris–HCl pH 7.5; 0.15 M LiCl; 1 mM EDTA). After removing wash buffer B, the beads were resuspended in 10 μl H_2_O and incubated at 65 °C for 2 min. Subsequently, the tube was directly placed on the magnetic stand for 30 s, and the supernatant containing the poly(A)^+^ RNA was quickly transferred to a new tube (tube B) on ice. The beads were then resuspended in the solution from tube A before being rotated for 5 min at room temperature. The tube was placed on the magnetic stand for 30 s, and the supernatant was discarded. The Dynabeads were again washed twice with wash buffer B and resuspended in 10 μl H_2_O. Following a 2-min incubation at 65 °C, the supernatant was taken using the magnetic stand and quickly transferred to tube B. The concentration of the isolated poly(A)^+^ RNA in tube B was determined with an Epoch microplate spectrophotometer.

First-strand cDNA synthesis was conducted with 100 ng of poly(A)^+^ RNA supplemented with 1 µl 100 µM Oligo(dT)_20_ primer, 2 µl 10 mM dNTP solution mix (New England Biolabs), and H_2_O to a final volume of 27 µl. Following 5 min at 65 °C, the samples were placed on ice, and 8 µl 5 × SSIV buffer (Thermo Fisher Scientific), 2 μl 100 mM 1,4-dithiothreitol (DTT; Thermo Fisher Scientific), 2 µl RNaseOUT recombinant ribonuclease inhibitor (Thermo Fisher Scientific), and 1 µl Superscript IV reverse transcriptase (Thermo Fisher Scientific) were added. Synthesis of cDNA was carried out at 52 °C for 50 min, followed by 10 min at 80 °C.

### Reverse transcription PCR

To analyze SNMP1-encoding nucleotide sequences in antennal cDNA of adult WT and SNMP1^−/−^ animals, the forward primer 5′-TACAAGCAGAAGGTGAAGCTGCGG (matching exon 3 of the SNMP1 gene) and the reverse primer 5′-TCCGCCTTGCGCTCCCAGTTCGCG (matching exon 6 of the gene encoding SNMP1) were used. PCR reactions were prepared utilizing 42 μl H_2_O, 5 μl 10 × Titanium Taq PCR buffer, 1 μl 10 mM dNTP solution mix, 0.5 μl 100 μM forward primer, 0.5 μl 100 μM reverse primer, 1 μl antennal cDNA, and 0.5 μl 50 × Titanium Taq DNA polymerase. Thermocycling conditions were identical to those described above for the PCR amplification of genomic DNA. An aliquot of the PCR product was analyzed by gel electrophoresis, and the rest was purified using the Monarch PCR & DNA cleanup kit as recommended by the manufacturer (New England Biolabs). The purified amplicon was finally eluted with 20 µl of H_2_O and sent for sequencing to Microsynth.

### Quantitative PCR

To amplify sequences coding for SNMP1 in quantitative PCR reactions, the forward primer 5′-CTCGCACTGGAGGAGAAGTACGTG and the reverse primer 5′-GGAGTGTCCAGGGCTAGTATCTG were used, resulting in a PCR fragment of 256 bp. For the amplification of the housekeeping gene GAPDH (GenBank: KU251430.1) that served as reference gene, the forward primer 5′-ATTGTTGAAGGTCTGATGACAACAG and the reverse primer 5′-TCCAGTTGATGCTGGAATAATGTTC were applied, leading to an amplicon of 230 bp. GAPDH is a useful reference gene for the normalization of expression in tissue from adult *S. gregaria* [53].

PCR reactions were conducted in a 10-μl reaction volume with 5 µl of Powerup SYBR green master mix (Thermo Fisher Scientific) and 3 µl of the cDNA template to which sense and antisense primers as well as H_2_O were added. Reactions were run in duplicate on a Quantstudio 3 real-time PCR system (Thermo Fisher Scientific) utilizing the following thermal cycling profile: 50 °C (for 2 min), 95 °C (for 2 min), followed by 40 steps of 95 °C for 15 s, 57 °C for 15 s, and 72 °C for 1 min. Subsequently, melting curve analyses were performed by running samples with the dissociation protocol (95 °C for 1 min, 60 °C for 1 min, and 95 °C for 15 s) to assess that quantitative PCR assays have only produced a single amplicon and that primer dimers have not been formed. In all quantitative PCR experiments, negative controls (H_2_0 was added instead of cDNA) were routinely run, and in these samples, no amplification of the fluorescence signal was observed. Raw data were recorded and analyzed with the Quantstudio design and analysis software v1.5.1 (Thermo Fisher Scientific) and were exported to Microsoft Excel for further evaluation.

In initial experiments with serial (10 ×) dilutions of antennal cDNA, standard curves for the gene transcripts were generated. Using different primer concentrations, it turned out that for SNMP1 and GAPDH, a 200 nM concentration of the sense and the antisense primer led to optimal values for PCR efficiency (E) and the coefficient of determination (R^2^).

Based on the CT values determined in quantitative PCR experiments, ∆CT values (∆CT = CT_SNMP1_ − CT_GAPDH_; [72]) and gene expression fold changes (relative expression) were calculated. For determining the relative expression, the method established by Pfaffl [54] was used since it provides efficiency correction. Two-tailed *p*-values for ∆CT and the relative expression were calculated with GraphPad Software (San Diego, CA, USA) using an unpaired *t*-test (https://www.graphpad.com/quickcalcs/ttest1/). For the relative expression, the determined values were compared to 1 (i.e., no change in expression).

### Measuring the length of antennae

The right antennae of 10 randomly selected adult males of both the WT and the SNMP1^−/−^ strain were cut off directly at the base of the antenna and the length was determined. The statistical analysis was performed using a two-sided Wilcoxon rank-sum test (https://www.statskingdom.com/170median_mann_whitney.html).

### Preparation of pipettes for stimulation in EAG experiments

PAN (purity 98%; Sigma-Aldrich) was dissolved in different concentrations in the solvent n-hexane (purity 95%; Applichem, Darmstadt, Germany). Linalool (LIN) (purity 97%; order number: L2602; Sigma-Aldrich) was diluted in dimethyl sulfoxide (DMSO; purity ≥ 99.5%; Sigma-Aldrich). In order to apply defined chemical stimuli in EAG experiments, the tip of glass Pasteur pipettes (150 mm; Neolab Migge, Heidelberg, Germany) was sealed with Parafilm M (Sigma-Aldrich) before inserting a round Whatman 40 filter paper (Sigma-Aldrich) on which 10 µl of the PAN- or LIN-containing solution or the solvent was pipetted. Thus, filter papers were loaded with 1, 20, 50, 75, or 100 µg of PAN or 1, 50, or 100 µg of LIN. After letting the solvent evaporate for 1 min, pipettes were sealed at their end with Parafilm M (Sigma-Aldrich).

### EAG recordings

For the EAG recordings, the measurements were carried out with technical equipment from Syntech (Kirchzarten, Germany) following the manufacturer’s recommendations (https://www.ockenfels-syntech.com/wp-content/uploads/EAGpract_man_fin) with slight modifications. In brief, the electrode holders were equipped with a 0.5-mm (diameter) silver electrode wire (order number: 265586; Sigma-Aldrich) chlorinated in 0.01 N HCl at 4.5 V directly before use. For preparing glass micropipettes, capillary tubes (GB150T-8P; Science Products, Hofheim, Germany) were pulled using the horizontal Pul-1 micropipette puller (settings: 1 for “delay” and 1 for “heat”), and the capillary tip was trimmed with scissors to an inner diameter that allowed insertion of the antennal base or tip. Glass micropipettes were filled with *Schistocerca* saline (9.82 g/l NaCl; 0.48 g/l KCl; 0.73 g/l MgCl_2_*6H_2_0; 0.47 g/l CaCl_2_*6H_2_O; 0.95 g/l NaH_2_P0_4_*2H_2_O; 0.18 g/l NaHCO_3_; pH 7.2 with NaOH; after [73]), assembled with the electrode holders and then with a Syntech micromanipulator and a Syntech EAG combi probe. The EAG combi probe was connected to the Syntech data acquisition controller IDAC-232, which was interconnected with a computer.

The right antenna of mature male locusts was excised and inserted between two glass micropipette electrodes. Using a Syntech CS-01 stimulus controller unit and a glassware humidifier, a continuous and humidified airstream of 7 ml/s was directed towards the antenna through a mixing tube, with the end of the tube placed 0.5 cm from the center of the antenna. For antennal stimulation, the Parafilm sealing was removed from a glass Pasteur pipette containing a round filter paper loaded with PAN or LIN solution or the solvent. The pipette tip was immediately inserted into a hole of the mixing tube to apply a 500-ms single puff into the airstream using the CS-01 stimulus controller. Each antenna was stimulated in a first series consecutively with the solvent and the different amounts of PAN or LIN in intervals of 1 min. In a second series, this procedure was repeated with the same antenna. The order of PAN or LIN stimuli was varied between different antennae. The antennal EAG responses were recorded using the Syntech EAG software. Raw data were exported to Microsoft Excel for further analysis. For each antenna, the measured value for the amplitude evoked by the solvent was subtracted from the amplitude values induced by the different PAN or LIN amounts. With the resulting values, for each amount of PAN or LIN, the mean was calculated based on the two technical replications. To analyze statistical significance, two-tailed *p*-values were determined with an unpaired *t*-test (https://www.graphpad.com/quickcalcs/ttest1/).

### Preparation of pipettes for stimulation in recordings with single sensilla

PAN was diluted in DMSO in different dilutions (1:10, 1:100, or 1:1000). Ten microliters of diluted PAN was loaded on a round piece of filter paper (~ 1 cm^2^) that was inserted in a glass Pasteur pipette. For stimulation with only the solvent, 10 µl of DMSO was applied to the filter paper.

### SSR experiments

In essence, SSR were conducted as recently described for migratory locusts [11]. Mature male desert locusts were inserted into 5-ml pipette tips, the point of which had previously been cut off to allow the head and the antennae to protrude out of the tips. Dental wax was used to immobilize the abdomen and the head of locusts in the tip as well as for attaching the tip, the head (the dorsal side downwards), and the antennae to a glass coverslip. Electrolytically sharpened tungsten wires served as electrodes, with the reference electrode inserted into the eye. Upon inspection with a microscope (BX51WI; Olympus, Shinjuku, Japan), the recording electrode was inserted into the base of a basiconic sensillum. Extracellular electrical signals were recorded, amplified (Syntech Universal AC/DC Probe; Syntech), sampled (96,000 samples/s), and band-pass filtered (300 to 3000 Hz with 50/60-Hz suppression) via a Syntech IDAC-4 acquisition controller connected to a computer. Spikes were extracted using version 3.7 of AutoSpike software (Syntech). For the detection of spikes with the AutoSpike software, the default settings of the program were used (detection time of 0.5 to 3 ms and a minimum amplitude of 10). Only spikes that were detected using these parameters were considered for further analysis.

Stimuli (PAN or the solvent DMSO) were delivered by inserting the tip of the pipette into a humidified and constant airstream flowing at 800 ml/min through an 8-mm inner diameter stainless steel tube ending 1 cm from the antenna. A 0.5-s single puff was applied into the airstream using a CS-55 stimulus controller (Syntech). Neuronal responses from basiconic OSNs were recorded for 10 s, starting 3 s prior to the stimulation period of 0.5 s. Basiconic sensilla were stimulated with DMSO and subsequently with PAN. For the calculation of PAN-elicited responses, only the spike rates 0.55 s before and 0.55 s after the onset of stimulation were considered. To calculate the PAN-induced spike rate, the spike rate before PAN stimulation (0.55 -s window) was first subtracted from the spike rate after PAN stimulation (0.55 -s window). Next, the DMSO-induced spike rate was determined by subtracting the spike rate before DMSO stimulation (0.55 -s window) from the spike rate after DMSO stimulation (0.55 -s window). Finally, the corrected PAN-induced spike rate was calculated by subtracting the DMSO-evoked spike rate from the PAN-induced spike rate. These corrected responses were multiplied by 1.81 to obtain the number of spikes per second. With the resulting values, the mean was calculated for each PAN concentration tested. To analyze statistical significance, two-tailed *p*-values were determined with an unpaired *t*-test (https://www.graphpad.com/quickcalcs/ttest1/).

### Preparation for functional calcium imaging of PNs

Calcium imaging was conducted following a recently established protocol and analysis pipeline to assess the responses of PNs in the AL upon odorant exposure [74]. In brief, PNs from sexually mature SNMP1^−/−^ and WT male locusts were retrogradely loaded with a dextran-conjugated calcium indicator (Cal-520-Dextran Conjugate MW 10,000; Biomol, Hamburg, Germany). For the procedure, a frontal incision was made between the compound eyes of an anesthetized and restrained locust to expose the brain around the injection site. Next, a crystal of the calcium indicator was injected into the medial calyx of the mushroom bodies using a glass capillary, and the head capsule was sealed with a drop of eicosane (Sigma-Aldrich) to prevent desiccation. After 12 h of incubation at 15 °C in a humid chamber, dye-injected specimens were prepared for imaging by removing the eicosane and by gently relocating the antennae ventrally to expose the AL. Finally, the recording site was covered with a translucent silicone (Kwik-Sil; World Precision Instruments).

### Functional confocal imaging and stimulation with odorants

Functional imaging was conducted with a laser scanning confocal microscope (LSM 510; Carl Zeiss Microscopy, Oberkochen, Germany) equipped with a Chameleon Ultra Ti:sapphire two-photon laser (Coherent, Santa Clara, CA, USA) and a water immersion objective (W Plan-Apochromat 20 × /1,0 DIC VIS-IR; Carl Zeiss Microscopy). Optical sections of the AL, centered around the layer harboring most of the somata of the PNs (approximately 35 µm beneath the surface of the AL), were acquired at 1 frame per second (fps) with a spatial resolution of 0.77 μm/px (*x*, *y*) and a full-width half maximum of 25 μm (*z*). For odorant stimulation, the ipsilateral antenna of tested preparations was placed inside the outlet (Teflon tube with an inner diameter of 0.87 mm) of a custom-built olfactometer [75]. The olfactometer controlled valves that diverted the flow to either a vial containing 200 μl of a diluted odorant or to clean air. Each individual was tested with PAN and LIN (both in mineral oil at dilutions of 1:10 and 1:100) and with the solvent (mineral oil) in a pseudo-random order. Stimulus pulses were 2 s long, and consecutive stimuli were separated by 2-min intervals. Odorants were prepared daily before the experiments and checked for consistency by a photoionization detector (Mini-PID model 200A; Aurora Scientific, Aurora, Ontario, Canada).

### Analysis of functional imaging data

Acquired imaging sequences (3D stack of images x–y-time) were processed and analyzed via a custom-written Matlab code (R2023a; The MathWorks, Natick, MA, USA), as previously described [74]. In brief, after preprocessing to reduce photon noise, motion, and bleaching corrections, stacks of the same stimulus set were prepared for analysis. Data were filtered by a 3-frame box-shaped kernel in the temporal domain, and the relative change in fluorescence (ΔF/F_0_ = (F − F_0_)/F_0_) was calculated with F_0_ being the mean pre-stimulus baseline activity during the 4 s prior to the stimulus. Finally, the response to the solvent was subtracted from each stack. Using a customized iterative segmentation algorithm (CalciSeg; https://github.com/YannickGuenzel/CalciSeg; [74]), we automatically segmented the AL of each preparation into regions of activity representing individual PNs (regions where activity changes similarly over time and across stimuli). Upon successful completion of the procedure, all valid regions (PNs) were automatically classified as 'responding' or 'non-responding' to each stimulus using Otsu's method for each mean intensity projection [76]. For statistical analyses of calcium imaging experiments, *p*-values were determined either by a paired two-sided Wilcoxon signed rank test using the signrank function of the Matlab software or by an unpaired two-sided Wilcoxon rank-sum test utilizing the ranksum function of the Matlab software.

### Re-pairing and mate choice assays

Behavioral experiments were performed similar to previously published protocols [13] using sexually mature locusts in small observation cages (10 × 10 × 20 cm). Experiments were conducted between noon and 6 pm.

In re-pairing assays, a pair of WT or SNMP1^−/−^ locusts was placed in a cage. For the experimental group, pairs were separated before 1 µl of 10% PAN [v/v in dichloromethane (CH_2_Cl_2_); Applichem] was applied to the pronotum of the females. Females of the control group were also segregated but received no treatment. After reunification, the re-pairing status was monitored every 30 min. Observations were terminated after 120 min. Similarly, a further control experiment using only pairs of WT locusts was conducted to assess potential effects of the solvent CH_2_Cl_2_ on re-pairing. In the latter approach, females were either painted with 1 µl CH_2_Cl_2_ or left untreated. For re-pairing assays, the percentage of couples that re-paired was determined. To calculate *p*-values (both tails), absolute numbers (i.e., raw numbers) of re-paired and unpaired couples were used in a Fisher's exact test (https://quantpsy.org/fisher/fisher.htm).

In mate choice assays, to a pair of WT or SNMP1^−/−^ locusts, another female (♀2) from a second pair of the same genotype was added. The original female (♀1) was treated with 1 µl of 1% or 10% PAN (v/v in CH_2_Cl_2_) and then returned to the cage. The second female (♀2) remained untreated; only its wings were slightly clipped to distinguish it from the original female. Assays were run until the male paired with one of the two females. If no pairing occurred within 30 min, the observations were terminated to prevent the experiments from being affected by the evaporation (i.e., decrease in concentration) of PAN. The *p*-values were calculated utilizing a calculator for a chi-square goodness of fit test (https://www.socscistatistics.com/tests/goodnessoffit/default2.aspx). In this test, the absolute numbers (raw numbers) of males choosing either ♀1 or ♀2 were used. For the expected values, half of the total number of tested males was taken.

### Supplementary Information

Additional file 1: Fig. S1 Schematic of the SNMP1 gene in *S. gregaria*. Grey boxes represent the nine exons encoding the SNMP1 protein (for the first and the ninth exon, only the coding regions are depicted). Due to their length of up to ~33,000 bp, intron sequences (dashed lines) are not shown to scale. The genomic region in exon 4 corresponding to the guide RNA (orange) and PAM site (green) is highlighted. The scale bar denotes a fragment of 100 bp.

Additional file 2: Fig. S2 WT and SNMP1^-^^/^^-^ variants of the *S. gregaria* SNMP1 protein. While the WT SNMP1 protein comprises 513 amino acid residues, the mutant SNMP1^-^^/^^-^ variant includes only 227 amino acids. For the WT variant, the segment encoded by exon 4 is shown in blue, whereas the two predicted transmembrane domains are denoted in pink. In the truncated SNMP1^-^^/^^-^ variant, amino acids encoded by the region of exon 4 that is upstream of the 22bp deletion are given in blue, while the remaining amino acids encoded by exon 4 are indicated in red (the sequence of the latter is not identical to the amino acid sequence of the WT variant). For the SNMP1^-^^/-^ protein, the amino acid residues encoded by exon 5 are marked in purple, and the single predicted transmembrane domain is highlighted in pink.

Additional file 3: Expression of mRNA for SNMP1 in the antenna of WT and SNMP1^-/-^ desert locusts. Following PCR approaches with a sense primer matching exon 3 and a reverse primer matching exon 6 of the SNMP1 gene, amplicons of the expected molecular size (~650 bp) were obtained using antennal cDNA from female WT and mutant animals as template. With these primers, no PCR product of the expected size was detectable when the cDNA template was omitted (con.). For the cDNA of mutants, PCR amplification was very faint (red ellipse). In the left lane, a DNA molecular size marker (M) was loaded (100 bp DNA Ladder; New England Biolabs). The numbers to the left indicate the molecular size in bp.

Additional file 4: Tab. S1 Length of antennae in adult male desert locusts of the WT and the SNMP1^-/-^strain

Additional file 5: Fig. S4 Representative traces of EAG recordings with antennae from WT and SNMP1^-/-^ males that were stimulated with PAN (100 µg). The bars above the traces indicate the stimulus time.

Additional file 6: Fig. S5 LIN-induced EAG responses in the antennae of adult WT and SNMP1^-^^/-^ males. The bars represent mean EAG responses to LIN (1, 50, or 100 µg), recorded from antennae of 14 WT (dark bars) and 14 mutant (light gray bars) males (only one antenna was measured per individual). The standard error of the mean (error bars) and two-tailed *p*-values (unpaired t-test) were calculated. The *p*-values are 0.4932 (1 µg), 0.4678 (50 µg), and 0.5782 (100 µg). The dataset for the EAG recordings with LIN is listed in Additional file 16: Tab. S8.

Additional file 7: Fig. S6 Single traces of SSR experiments using basiconic sensilla stimulated with PAN (1:100 dilution). The bars above the traces denote the stimulus time. Traces are from male WT and SNMP1^-^^/-^ animals, respectively.

Additional file 8: Fig. S7 The solvent dichloromethane (CH_2_Cl_2_) does not affect re-pairing behavior in *S. gregaria*. Couples of WT desert locusts were placed individually in separate cages. Females were briefly taken out of the cage, and their pronotum was either painted with 1 µl of CH_2_Cl_2_ (shaded bars) or painting was omitted (dotted bars). Then, females were returned to the cage with the male, and re-pairing was recorded 30, 60, 90, and 120 min later. For each time point, the re-pairing rate was calculated as 100% × the number of re-paired couples divided by the total number of couples. The data depicted are based on 20 couples for which females were painted with CH_2_Cl_2_ and 20 couples with females remaining unpainted. *P*-values (both tails) were calculated using Fisher's exact test. The *p*-values were >0.99 for all tested time points. The dataset of the re-pairing assays with and without CH_2_Cl_2_ is shown in Additional file 17: Tab. S9.

Additional file 9: Fig. S8 Original SDS-PAGE and Western blot analyses. SDS-PAGE (stained with Coomassie blue, upper panel) and Western blot analysis (lower panel) were conducted with 5, 10, or 15 µg of protein fractions from the antennae of WT or SNMP1^-/-^ (KO) animals (males and females). Immunodetection was carried out with the antibody against SNMP1 that labeled a band with the predicted molecular mass of SNMP1 (~57 kDA) only in the protein fraction of WT desert locusts. The molecular mass and position of a molecular weight marker (M; PageRuler prestained protein ladder; Thermo Fisher Scientific) used in the SDS-PAGE and the Western blot analysis are denoted on the left.

Additional file 10: Tab. S2. Dataset of the quantitative PCR experiments

Additional file 11: Tab. S3. Dataset of the EAG recordings with PAN

Additional file 12:Tab. S4. Dataset of the SSR experiments with PAN

Additional file 13: Tab. S5. Datasets of the calcium imaging experiments

Additional file 14: Tab. S6. Dataset of the re-pairing assays with PAN

Additional file 15: Tab. S7. Dataset of the mate choice assays

Additional file 16: Tab. S8. Dataset for the EAG recordings with LIN

Additional file 17: Tab. S9. Dataset of the re-pairing assays with CH_2_Cl_2_

## Data Availability

All data generated or analyzed during this study are included in this published article and its supplementary information files. The raw data used for the statistical analyses throughout the manuscript are included in Additional files 10 to 17.

## Abbreviations

AL: Antennal lobe
bp: Base pairs
CT: Threshold cycle
cVA: Cis-vaccenyl acetate
dNTP: 2'-Deoxynucleoside 5'-triphosphate
EAG: Electroantennography
GAPDH: Glyceraldehyde-3-phosphate dehydrogenase
LIN: Linalool
ORs: Odorant receptors
OSNs: Olfactory sensory neurons
PAM: Protospacer adjacent motif
PAN: Phenylacetonitrile
PNs: Projection neurons
SNMP1: Sensory neuron membrane protein 1
SNMP1^−/−^: SNMP1-deficient desert locust line
SSR: Single sensillum recording(s)
WT: Wild-type

## References

[1] Simpson SJ, Sword GA (2008). Locusts. Curr Biol.

[2] van Huis A, Cressman K, Magor JI (2007). Preventing desert locust plagues: optimizing management interventions. Entomol Exp Appl.

[3] Pener MP, Simpson SJ: Locust phase polyphenism: an update. In: Advances in Insect Physiology*.* Edited by Simpson SJ, Pener MP, vol. 36: Academic Press; 2009: 1–272.

[4] Hassanali A, Njagi PG, Bashir MO (2005). Chemical ecology of locusts and related acridids. Annu Rev Entomol.

[5] Applebaum SW, Heifetz Y (1999). Density-dependent physiological phase in insects. Annu Rev Entomol.

[6] Roessingh P, Bouaïchi A, Simpson SJ (1998). Effects of sensory stimuli on the behavioural phase state of the desert locust. Schistocerca gregaria J Insect Physiol.

[7] Ferenz HJ, Seidelmann K (2003). Pheromones in relation to aggregation and reproduction in desert locusts. Physiol Entomol.

[8] Obeng-Ofori D, Torto B, Njagi PG, Hassanali A, Amiani H (1994). Fecal volatiles as part of the aggregation pheromone complex of the desert locust, *Schistocerca gregaria* (Forskal) (Orthoptera: Acrididae). J Chem Ecol.

[9] Dillon RJ, Vennard CT, Charnley AK (2000). Exploitation of gut bacteria in the locust. Nature.

[10] Fuzeau-Braesch S, Genin E, Jullien R, Knowles E, Papin C (1988). Composition and role of volatile substances in atmosphere surrounding two gregarious locusts, *Locusta migratoria* and *Schistocerca gregaria*. J Chem Ecol.

[11] Chang HT, Cassau S, Krieger J, Guo XJ, Knaden M, Kang L, Hansson BS (2023). A chemical defense deters cannibalism in migratory locusts. Science.

[12] Seidelmann K, Warnstorff K, Ferenz HJ (2005). Phenylacetonitrile is a male specific repellent in gregarious desert locusts. Schistocerca gregaria Chemoecology.

[13] Seidelmann K, Ferenz HJ (2002). Courtship inhibition pheromone in desert locusts. Schistocerca gregaria J Insect Physiol.

[14] Seidelmann K, Luber K, Ferenz HJ (2000). Analysis of release and role of benzyl cyanide in male desert locusts. Schistocerca gregaria J Chem Ecol.

[15] Ochieng SA, Hansson BS (1999). Responses of olfactory receptor neurones to behaviourally important odours in gregarious and solitarious desert locust. Schistocerca gregaria Physiol Entomol.

[16] Cui X, Wu C, Zhang L (2011). Electrophysiological response patterns of 16 olfactory neurons from the trichoid sensilla to odorant from fecal volatiles in the locust, *Locusta migratoria manilensis*. Arch Insect Biochem Physiol.

[17] Ochieng SA, Hallberg E, Hansson BS (1998). Fine structure and distribution of antennal sensilla of the desert locust, *Schistocerca gregaria* (Orthoptera: Acrididae). Cell Tissue Res.

[18] Anton S, Hansson BS (1996). Antennal lobe interneurons in the desert locust *Schistocerca gregaria* (Forskal): processing of aggregation pheromones in adult males and females. J Comp Neurol.

[19] Hansson BS, Occhieng SA, Grosmaitre X, Anton S, Njagi PGN (1996). Physiologicsal responses and central nervous projections of antennal olfactory receptor neurons in the adult desert locust, *Schistocerca gregaria* (Orthoptera: Acrididae). J Comp Physiol A.

[20] Strausfeld NJ, Hansen L, Li YS, Gomez RS, Ito K (1998). Evolution, discovery, and interpretations of arthropod mushroom bodies. Learn Mem.

[21] Heisenberg M (1998). What do the mushroom bodies do for the insect brain?. An introduction Learn Mem.

[22] Ernst KD, Boeckh J, Boeckh V (1977). A neuroanatomical study on the organization of the central antennal pathways in insects. Cell Tissue Res.

[23] Ignell R, Anton S, Hansson BS (1998). Central nervous processing of behaviourally relevant odours in solitary and gregarious fifth instar locusts. Schistocerca gregaria J Comp Physiol A.

[24] Ignell R, Anton S, Hansson BS (2001). The antennal lobe of orthoptera - Anatomy and evolution. Brain Behav Evolut.

[25] Hansson BS, Anton S (2000). Function and morphology of the antennal lobe: new developments. Annu Rev Entomol.

[26] Fleischer J, Pregitzer P, Breer H, Krieger J (2018). Access to the odor world: olfactory receptors and their role for signal transduction in insects. Cell Mol Life Sci.

[27] Wicher D, Miazzi F (2021). Functional properties of insect olfactory receptors: ionotropic receptors and odorant receptors. Cell Tissue Res.

[28] Benton R, Vannice KS, Gomez-Diaz C, Vosshall LB (2009). Variant ionotropic glutamate receptors as chemosensory receptors in *Drosophila*. Cell.

[29] Vosshall LB, Amrein H, Morozov PS, Rzhetsky A, Axel R (1999). A spatial map of olfactory receptor expression in the *Drosophila* antenna. Cell.

[30] Clyne PJ, Warr CG, Freeman MR, Lessing D, Kim J, Carlson JR (1999). A novel family of divergent seven-transmembrane proteins: candidate odorant receptors in *Drosophila*. Neuron.

[31] Larsson MC, Domingos AI, Jones WD, Chiappe ME, Amrein H, Vosshall LB (2004). Or83b encodes a broadly expressed odorant receptor essential for *Drosophila* olfaction. Neuron.

[32] Sato K, Pellegrino M, Nakagawa T, Nakagawa T, Vosshall LB, Touhara K (2008). Insect olfactory receptors are heteromeric ligand-gated ion channels. Nature.

[33] Wicher D, Schafer R, Bauernfeind R, Stensmyr MC, Heller R, Heinemann SH, Hansson BS (2008). *Drosophila* odorant receptors are both ligand-gated and cyclic-nucleotide-activated cation channels. Nature.

[34] Benton R, Sachse S, Michnick SW, Vosshall LB (2006). Atypical membrane topology and heteromeric function of *Drosophila* odorant receptors in vivo. PLoS Biol.

[35] Benton R, Vannice KS, Vosshall LB (2007). An essential role for a CD36-related receptor in pheromone detection in *Drosophila*. Nature.

[36] Gomez-Diaz C, Bargeton B, Abuin L, Bukar N, Reina JH, Bartoi T, Graf M, Ong H, Ulbrich MH, Masson JF (2016). A CD36 ectodomain mediates insect pheromone detection via a putative tunnelling mechanism. Nat Commun.

[37] Jin X, Ha TS, Smith DP (2008). SNMP is a signaling component required for pheromone sensitivity in *Drosophila*. Proc Natl Acad Sci U S A.

[38] Liu S, Chang H, Liu W, Cui W, Liu Y, Wang Y, Ren B, Wang G (2020). Essential role for SNMP1 in detection of sex pheromones in *Helicoverpa armigera*. Insect Biochem Mol Biol..

[39] Zhang HJ, Xu W, Chen QM, Sun LN, Anderson A, Xia QY, Papanicolaou A (2020). A phylogenomics approach to characterizing sensory neuron membrane proteins (SNMPs) in Lepidoptera. Insect Biochem Mol Biol.

[40] Rogers ME, Sun M, Lerner MR, Vogt RG (1997). Snmp-1, a novel membrane protein of olfactory neurons of the silk moth *Antheraea polyphemus* with homology to the CD36 family of membrane proteins. J Biol Chem.

[41] Rogers ME, Krieger J, Vogt RG (2001). Antennal SNMPs (sensory neuron membrane proteins) of Lepidoptera define a unique family of invertebrate CD36-like proteins. J Neurobiol.

[42] Rogers ME, Steinbrecht RA, Vogt RG (2001). Expression of SNMP-1 in olfactory neurons and sensilla of male and female antennae of the silkmoth *Antheraea polyphemus*. Cell Tissue Res.

[43] Silverstein RL, Febbraio M (2009). CD36, a scavenger receptor involved in immunity, metabolism, angiogenesis, and behavior. Sci Signal.

[44] Pepino MY, Kuda O, Samovski D, Abumrad NA (2014). Structure-function of CD36 and importance of fatty acid signal transduction in fat metabolism. Annu Rev Nutr.

[45] Glatz JFC, Nabben M, Luiken J (2022). CD36 (SR-B2) as master regulator of cellular fatty acid homeostasis. Curr Opin Lipidol.

[46] Chen Y, Zhang J, Cui W, Silverstein RL (2022). CD36, a signaling receptor and fatty acid transporter that regulates immune cell metabolism and fate. J Exp Med.

[47] Pregitzer P, Greschista M, Breer H, Krieger J (2014). The sensory neurone membrane protein SNMP1 contributes to the sensitivity of a pheromone detection system. Insect Mol Biol.

[48] Jiang X, Pregitzer P, Grosse-Wilde E, Breer H, Krieger J (2016). Identification and characterization of two "Sensory Neuron Membrane Proteins" (SNMPs) of the desert locust, *Schistocerca gregaria* (Orthoptera: Acrididae). J Insect Sci.

[49] Cassau S, Sander D, Karcher T, Laue M, Hause G, Breer H, Krieger J (2022). The sensilla-specific expression and subcellular localization of SNMP1 and SNMP2 reveal novel insights into their roles in the antenna of the desert locust *Schistocerca gregaria*. Insects..

[50] Pregitzer P, Jiang X, Grosse-Wilde E, Breer H, Krieger J, Fleischer J (2017). In search for pheromone receptors: certain members of the odorant receptor family in the desert locust *Schistocerca gregaria* (Orthoptera: Acrididae) are co-expressed with SNMP1. Int J Biol Sci.

[51] Pregitzer P, Jiang X, Lemke RS, Krieger J, Fleischer J, Breer H (2019). A subset of odorant receptors from the desert locust *Schistocerca gregaria* is co-expressed with the Sensory Neuron Membrane Protein 1. Insects.

[52] Carroll D (2014). Genome engineering with targetable nucleases. Annu Rev Biochem.

[53] Van Hiel MB, Van Wielendaele P, Temmerman L, Van Soest S, Vuerinckx K, Huybrechts R, Broeck JV, Simonet G (2009). Identification and validation of housekeeping genes in brains of the desert locust *Schistocerca gregaria* under different developmental conditions. BMC Mol Biol.

[54] Pfaffl MW (2001). A new mathematical model for relative quantification in real-time RT-PCR. Nucleic Acids Res.

[55] Olsson SB, Hansson BS: Electroantennogram and single sensillum recording in insect antennae. In: Pheromone Signaling Methods in Molecular Biology*.* Edited by Touhara K, vol. 1068. Totowa, NJ: Humana Press; 2013:157–177. 10.1007/978-1-62703-619-1_11 24014360

[56] Roelofs WL, Hummel HE, Miller TA (1984). Electroantennogram assays: rapid and convenient screening procedures for pheromones. Techniques in Pheromone Research Springer Series in Experimental Entomology.

[57] Sachse S, Galizia CG (2002). Role of inhibition for temporal and spatial odor representation in olfactory output neurons: a calcium imaging study. J Neurophysiol.

[58] Günzel Y, McCollum J, Paoli M, Galizia CG, Petelski I, Couzin-Fuchs E (2021). Social modulation of individual preferences in cockroaches. iScience..

[59] Sun D, Guo Z, Liu Y, Zhang Y (2017). Progress and prospects of CRISPR/Cas systems in insects and other arthropods. Front Physiol..

[60] Singh S, Rahangdale S, Pandita S, Saxena G, Upadhyay SK, Mishra G, Verma PC (2022). CRISPR/Cas9 for insect pests management: a comprehensive review of advances and applications. Agriculture..

[61] Ramesh N, Khan Y (2021). Can CRISPR help control locust populations to reduce the impact of plague outbreaks?. Journal of Student Research..

[62] Li Y, Zhang J, Chen D, Yang P, Jiang F, Wang X, Kang L (2016). CRISPR/Cas9 in locusts: Successful establishment of an olfactory deficiency line by targeting the mutagenesis of an odorant receptor co-receptor (Orco). Insect Biochem Mol Biol.

[63] Ran FA, Hsu PD, Wright J, Agarwala V, Scott DA, Zhang F (2013). Genome engineering using the CRISPR-Cas9 system. Nat Protoc.

[64] Karousis ED, Nasif S, Muhlemann O (2016). Nonsense-mediated mRNA decay: novel mechanistic insights and biological impact. Wiley Interdiscip Rev RNA.

[65] Li Z, Ni JD, Huang J, Montell C (2014). Requirement for *Drosophila* SNMP1 for rapid activation and termination of pheromone-induced activity. PLoS Genet.

[66] Couto A, Alenius M, Dickson BJ (2005). Molecular, anatomical, and functional organization of the *Drosophila* olfactory system. Curr Biol.

[67] Fishilevich E, Vosshall LB (2005). Genetic and functional subdivision of the *Drosophila* antennal lobe. Curr Biol.

[68] Dweck HK, Ebrahim SA, Kromann S, Bown D, Hillbur Y, Sachse S, Hansson BS, Stensmyr MC (2013). Olfactory preference for egg laying on citrus substrates in *Drosophila*. Curr Biol.

[69] Verlinden H, Sterck L, Li J, Li Z, Yssel A, Gansemans Y, Verdonck R, Holtof M, Song H, Behmer ST et al. First draft genome assembly of the desert locust *Schistocerca gregaria*. F1000Res. 2020;9:775. 10.12688/f1000research.25148.1 PMC7607483 33163158

[70] Bae S, Park J, Kim JS (2014). Cas-OFFinder: a fast and versatile algorithm that searches for potential off-target sites of Cas9 RNA-guided endonucleases. Bioinformatics.

[71] Cassau S, Degen A, Kruger S, Krieger J (2023). The specific expression patterns of sensory neuron membrane proteins are retained throughout the development of the desert locust *Schistocerca gregaria*. Curr Res Insect Sci.

[72] Livak KJ, Schmittgen TD (2001). Analysis of relative gene expression data using real-time quantitative PCR and the 2(-Delta Delta C(T)) Method. Methods.

[73] Mordue W, Goldsworthy GJ (1969). The physiological effects of corpus cardiacum extracts in locusts. Gen Comp Endocrinol.

[74] Petelski I, Günzel Y, Sayin S, Kraus S, Couzin-Fuchs E (2024). Synergistic olfactory processing for social plasticity in desert locusts. Nat Commun..

[75] Raiser G, Galizia CG, Szyszka P (2017). A high-bandwidth dual-channel olfactory stimulator for studying temporal sensitivity of olfactory processing. Chem Senses.

[76] Otsu N (1979). Threshold selection method from gray-level histograms. IEEE Trans Syst Man Cybern.

