# Supplementary figures and images for: SNMP1 is critical for sensitive detection of the desert locust aromatic courtship inhibition pheromone phenylacetonitrile
Source: BMC Biol. 2024 Jul 8;22:150. doi: 10.1186/s12915-024-01941-x (PMC11229289; doi:10.1186/s12915-024-01941-x)

Additional file 1: Fig. S1

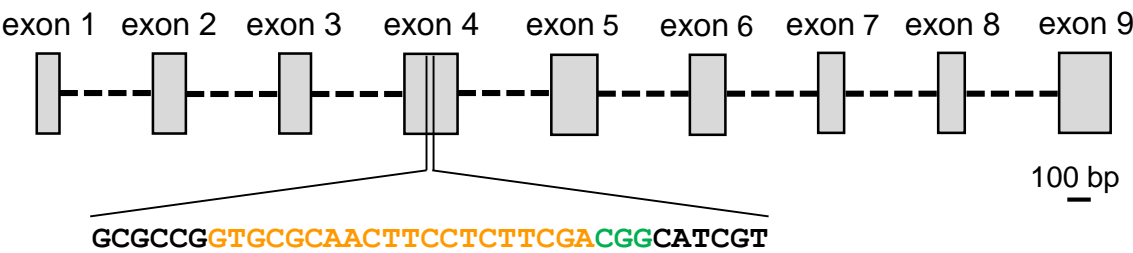

Supplement: Supplementary file 1 — Additional file 1: Fig. S1 Schematic of the SNMP1 gene in S. gregaria. Grey boxes represent the nine exons encoding the SNMP1 protein (for the first and the ninth exon, only the coding regions are depicted). Due to their length of up to ~33,000 bp, intron sequences (dashed lines) are not shown to scale. The genomic region in exon 4 corresponding to the guide RNA (orange) and PAM site (green) is highlighted. The scale bar denotes a fragment of 100 bp. [file 12915_2024_1941_MOESM1_ESM.pdf]

Additional file 3: Fig. S3

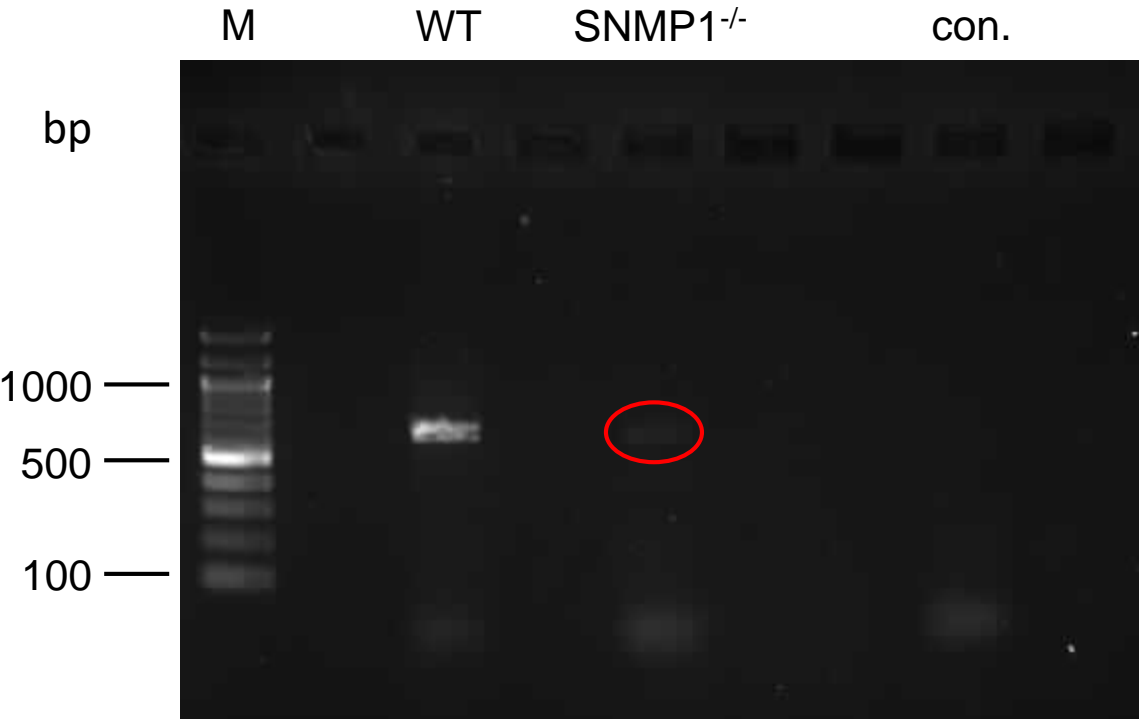

Supplement: Supplementary file 3 — Additional file 3: Expression of mRNA for SNMP1 in the antenna of WT and SNMP1-/- desert locusts. Following PCR approaches with a sense primer matching exon 3 and a reverse primer matching exon 6 of the SNMP1 gene, amplicons of the expected molecular size (~650 bp) were obtained using antennal cDNA from female WT and mutant animals as template. With these primers, no PCR product of the expected size was detectable when the cDNA template was omitted (con.). For the cDNA of mutants, PCR amplification was very faint (red ellipse). In the left lane, a DNA molecular size marker (M) was loaded (100 bp DNA Ladder; New England Biolabs). The numbers to the left indicate the molecular size in bp. [file 12915_2024_1941_MOESM3_ESM.pdf]

Additional file 5: Fig. S4

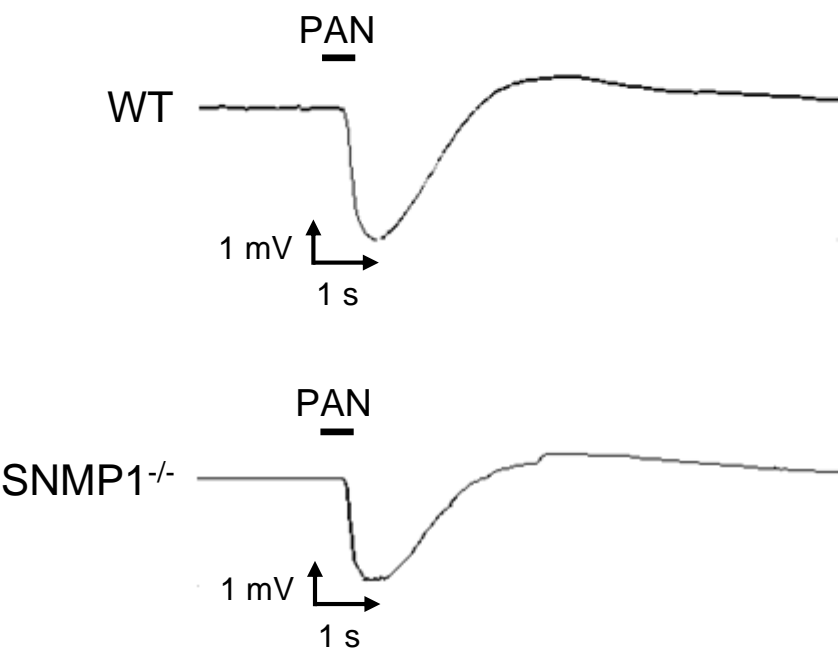

Supplement: Supplementary file 5 — Additional file 5: Fig. S4 Representative traces of EAG recordings with antennae from WT and SNMP1-/- males that were stimulated with PAN (100 µg). The bars above the traces indicate the stimulus time. [file 12915_2024_1941_MOESM5_ESM.pdf]

Additional file 6: Fig. S5

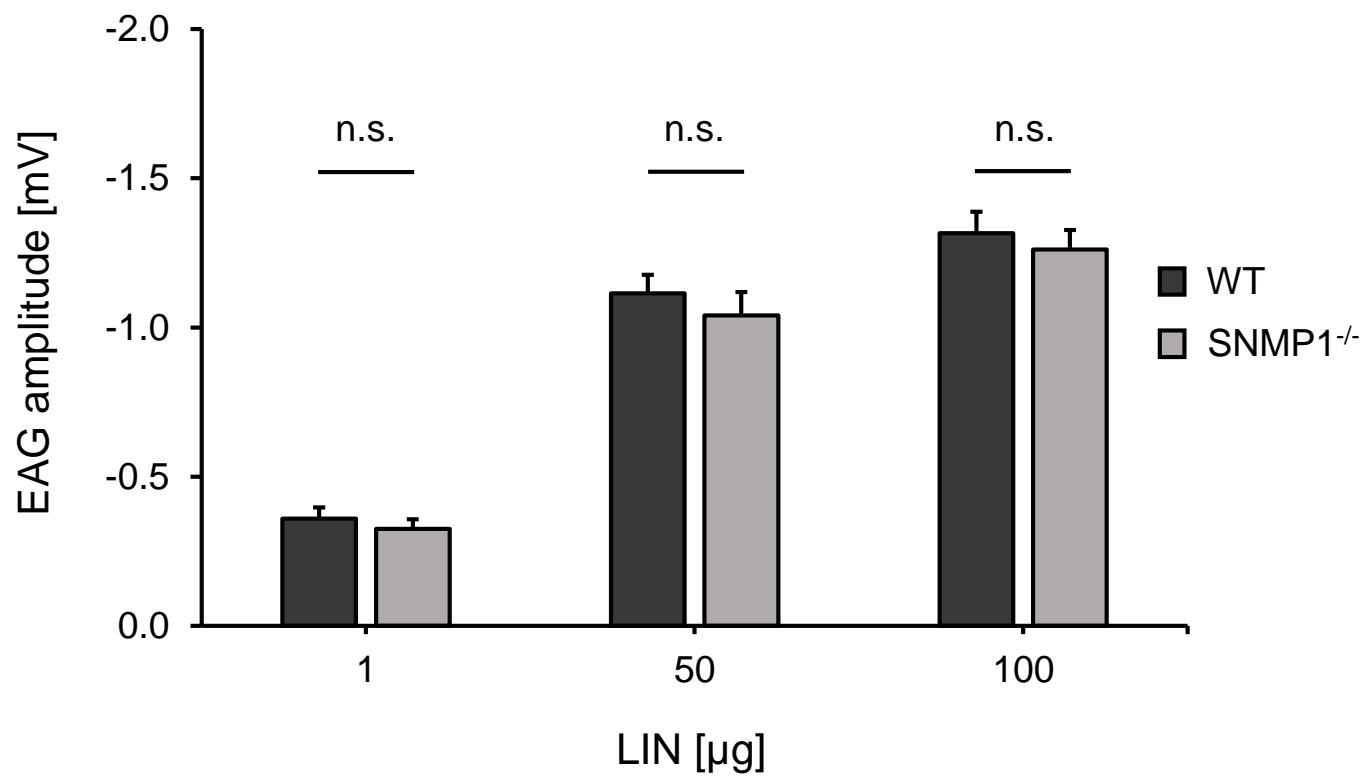

Supplement: Supplementary file 6 — Additional file 6: Fig. S5 LIN-induced EAG responses in the antennae of adult WT and SNMP1-/- males. The bars represent mean EAG responses to LIN (1, 50, or 100 µg), recorded from antennae of 14 WT (dark bars) and 14 mutant (light gray bars) males (only one antenna was measured per individual). The standard error of the mean (error bars) and two-tailed p-values (unpaired t-test) were calculated. The p-values are 0.4932 (1 µg), 0.4678 (50 µg), and 0.5782 (100 µg). The dataset for the EAG recordings with LIN is listed in Additional file 16: Tab. S8. [file 12915_2024_1941_MOESM6_ESM.pdf]

Additional file 7: Fig. S6

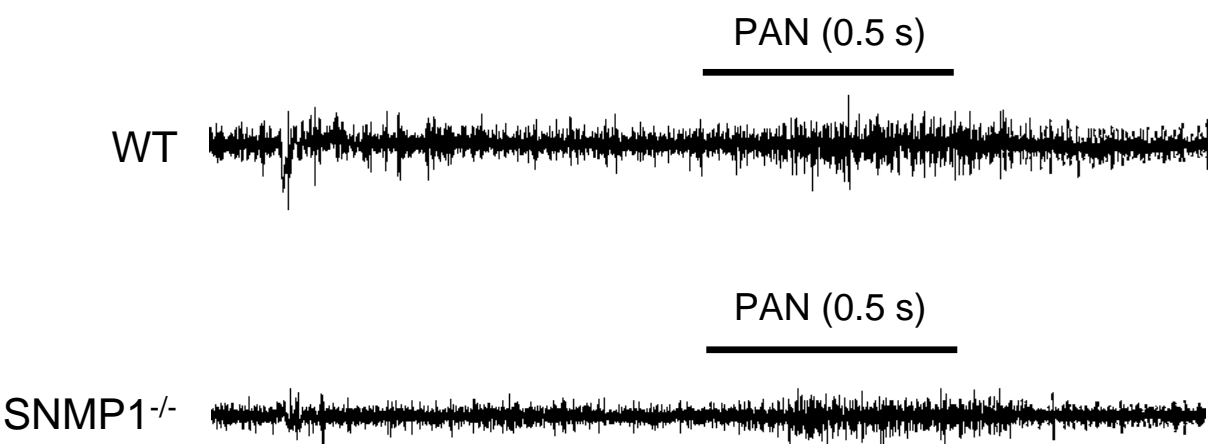

Supplement: Supplementary file 7 — Additional file 7: Fig. S6 Single traces of SSR experiments using basiconic sensilla stimulated with PAN (1:100 dilution). The bars above the traces denote the stimulus time. Traces are from male WT and SNMP1-/- animals, respectively. [file 12915_2024_1941_MOESM7_ESM.pdf]

Additional file 8: Fig. S7

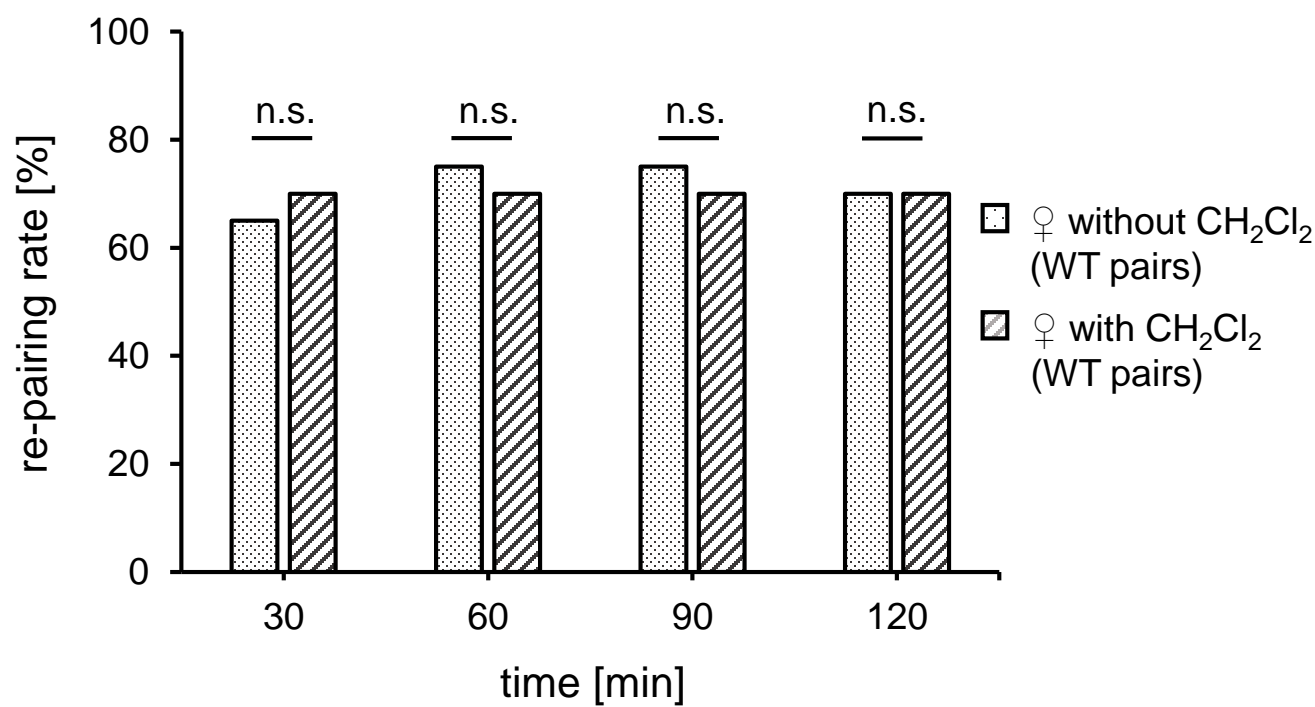

Supplement: Supplementary file 8 — Additional file 8: Fig. S7 The solvent dichloromethane (CH2Cl2) does not affect re-pairing behavior in S. gregaria. Couples of WT desert locusts were placed individually in separate cages. Females were briefly taken out of the cage, and their pronotum was either painted with 1 µl of CH2Cl2 (shaded bars) or painting was omitted (dotted bars). Then, females were returned to the cage with the male, and re-pairing was recorded 30, 60, 90, and 120 min later. For each time point, the re-pairing rate was calculated as 100% × the number of re-paired couples divided by the total number of couples. The data depicted are based on 20 couples for which females were painted with CH2Cl2 and 20 couples with females remaining unpainted. P-values (both tails) were calculated using Fisher's exact test. The p-values were >0.99 for all tested time points. The dataset of the re-pairing assays with and without CH2Cl2 is shown in Additional file 17: Tab. S9. [file 12915_2024_1941_MOESM8_ESM.pdf]

Additional file 9: Fig. S8

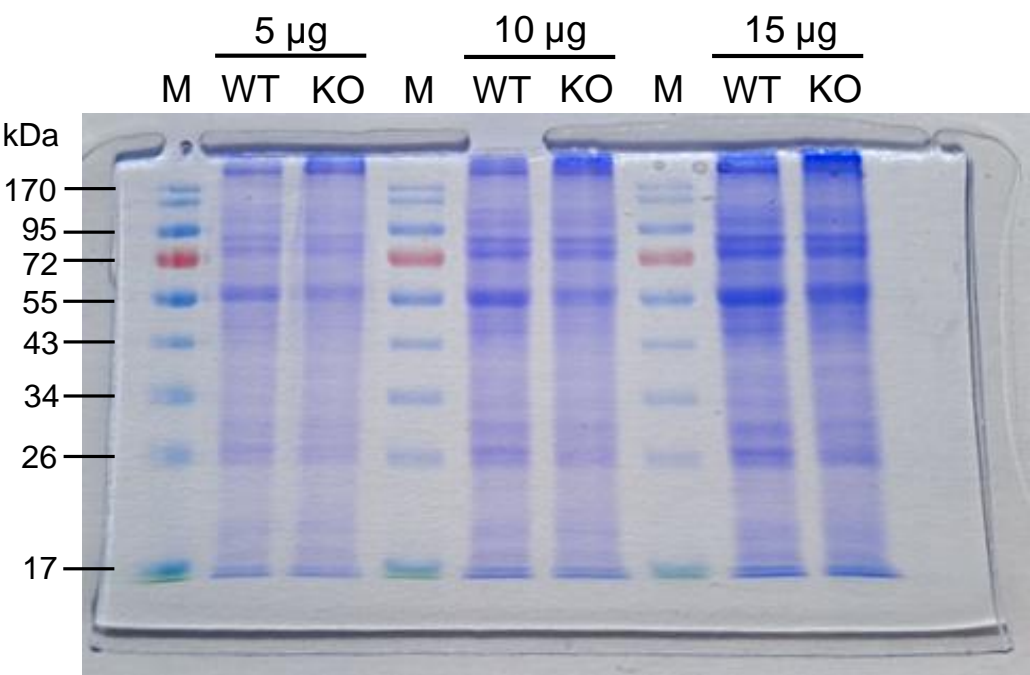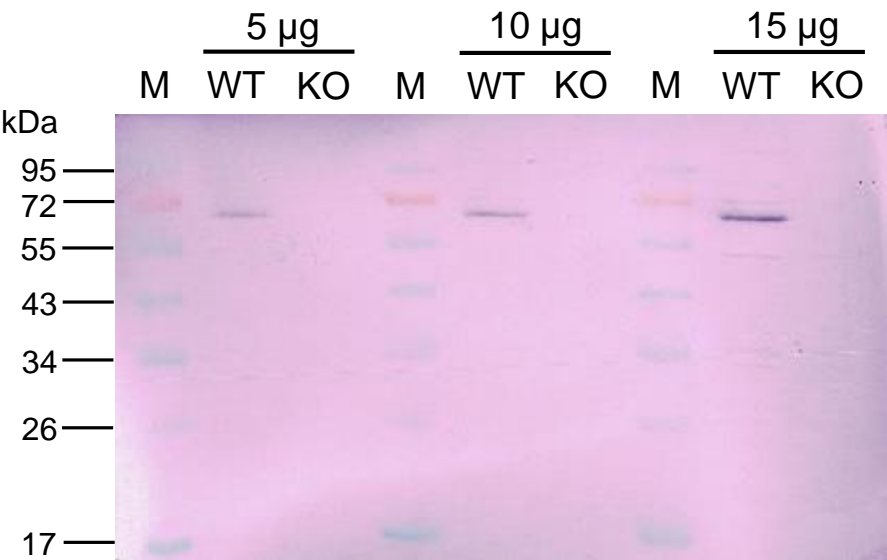

Supplement: Supplementary file 9 — Additional file 9: Fig. S8 Original SDS-PAGE and Western blot analyses. SDS-PAGE (stained with Coomassie blue, upper panel) and Western blot analysis (lower panel) were conducted with 5, 10, or 15 µg of protein fractions from the antennae of WT or SNMP1-/- (KO) animals (males and females). Immunodetection was carried out with the antibody against SNMP1 that labeled a band with the predicted molecular mass of SNMP1 (~57 kDA) only in the protein fraction of WT desert locusts. The molecular mass and position of a molecular weight marker (M; PageRuler prestained protein ladder; Thermo Fisher Scientific) used in the SDS-PAGE and the Western blot analysis are denoted on the left. [file 12915_2024_1941_MOESM9_ESM.pdf]
