# Supplementary material for: SNMP1 is critical for sensitive detection of the desert locust aromatic courtship inhibition pheromone phenylacetonitrile
Source: BMC Biol. 2024 Jul 8;22:150. doi: 10.1186/s12915-024-01941-x (PMC11229289; doi:10.1186/s12915-024-01941-x)
Supplement: Supplementary file 2 — Additional file 2: Fig. S2 WT and SNMP1-/- variants of the S. gregaria SNMP1 protein. While the WT SNMP1 protein comprises 513 amino acid residues, the mutant SNMP1-/- variant includes only 227 amino acids. For the WT variant, the segment encoded by exon 4 is shown in blue, whereas the two predicted transmembrane domains are denoted in pink. In the truncated SNMP1-/- variant, amino acids encoded by the region of exon 4 that is upstream of the 22bp deletion are given in blue, while the remaining amino acids encoded by exon 4 are indicated in red (the sequence of the latter is not identical to the amino acid sequence of the WT variant). For the SNMP1-/- protein, the amino acid residues encoded by exon 5 are marked in purple, and the single predicted transmembrane domain is highlighted in pink. [file 12915_2024_1941_MOESM2_ESM.pdf]

Additional file 2: Fig. S2

WT SNMP1

MQLPVGLAAGGGGVFFMAVVAGWYGMPKLISSQIASGLALKKGSDIRQMWSNFSDPIDFRVYV
LNLTNPEAVHRGEKPIVQEIGPYFYEEYKQKVKLRDHKEDDTVSYNNKITWLFNQGKSAPGLT
GDELVTLPHPLLLGLLLTLERDKPGMLALVNKAIPPLFRKPESIFVTAPVRNFLFDGIVINCT
VTDFSAKALCTGLKKEAKELKREGDNFFFSFFGHKNGTVDAGRLRVKRGIQNIDDLGRVVAFN
GEPKMSAWRGDPCNDLRGTDSTIFPPFRDPKEPIVAFGPDLCLSLGANWERKAEYMGVPGNRY
TAELPDMKGNPEHHCYCPTEQTCLEKGTLDLSPCAGAPVIATLPHFYLASETYLQTVSGLQPT
KENHELFMVFESTTGSPMEARKRLQFNMFLHKINKIDLLANVPYALMPLIWVEEGLALEEKYV
STLRMLFRMQGIMSGVKWTLMAVGMGMAGAGGYLHFKRRKELVVGPAEPKKVVAGHDTTGHPI
RLESSHSRY

SNMP1$^{-/-}$

MQLPVGLAAGGGGVFFMAVVAGWYGMPKLISSQIASGLALKKGSDIRQMWSNFSDPIDFRVYV
LNLTNPEAVHRGEKPIVQEIGPYFYEEYKQKVKLRDHKEDDTVSYNNKITWLFNQGKSAPGLT
GDELVTLPHPLLLGLLLTLERDKPGMLALVNKAIPPLFRKPESIFVTAPASSSTAPSPTSRRR
RSARASRRRPRSSSARATTSSSPSLDTKMARWTRGGCG
