# Supplementary material for: SNMP1 is critical for sensitive detection of the desert locust aromatic courtship inhibition pheromone phenylacetonitrile
Source: BMC Biol. 2024 Jul 8;22:150. doi: 10.1186/s12915-024-01941-x (PMC11229289; doi:10.1186/s12915-024-01941-x)
Supplement: Supplementary file 4 — Additional file 4: Tab. S1 Length of antennae in adult male desert locusts of the WT and the SNMP1-/-strain [file 12915_2024_1941_MOESM4_ESM.pdf]

Additional file 4: Tab. S1

Length of antennae in adult male desert locusts of the WT and the SNMP1$^{-/-}$ strain.

	WT	SNMP1$^{-/-}$
animal 1	1.3	1.2
animal 2	1.1	1.3
animal 3	1.2	1.1
animal 4	1.2	1.2
animal 5	1.1	1.2
animal 6	1.1	1.2
animal 7	1.2	1.3
animal 8	1.3	1.2
animal 9	1.3	1.3
animal 10	0.9	1.1
mean	1.17	1.21
standard error of the mean	0.04	0.023

The length of the right antenna from 10 randomly selected adult WT and SNMP1$^{-/-}$ males was determined and is given in centimeters. A p-value of 0.5491 was calculated by a two-tailed Wilcoxon rank-sum test.
